# Supplementary material for: A streamlined pipeline for multiplexed quantitative site-specific N-glycoproteomics
Source: Nat Commun. 2020 Oct 19;11:5268. doi: 10.1038/s41467-020-19052-w (PMC7572468; doi:10.1038/s41467-020-19052-w)
Supplement: Supplementary file 1 — Supplementary Information [file 41467_2020_19052_MOESM1_ESM.pdf]

A streamlined pipeline for multiplexed quantitative site-specific N-glycoproteomics

Pan *et al.*

Table of contents:

Supplementary Notes:

Supplementary note 1. Selection of quantification methods for intact glycopeptides.

Supplementary note 2. Development and optimization of sample preparation for quantitative glycoproteomics.

Supplementary note 3. Development of Glyco-SPS-MS3 for confident glycopeptide identification and accurate quantification.

Supplementary note 4. Development and usage of GlycoBinder.

Supplementary Figures:

Supplementary figure 1. Optimization of experimental parameters for sample preparation of TMT-labelled glycopeptides.

Supplementary figure 2. Effects of TMT-labelling on glycopeptide identification.

Supplementary figure 3. Fragmentation of TMT labelled N-glycopeptides under different HCD NCEs in LC-MS/MS analyses.

Supplementary figure 4. Kernel distributions of detected reporter ion intensities in glycopeptide or non-glycopeptide from previous publications.

Supplementary figure 5. Representative MS2 and MS3 spectra of a glycopeptide YKNNSDISSTR+Hex5HexNAc5Fuc acquired using standard HCD MS2 or Glyco-SPS-MS3.

Supplementary figure 6. Selection of MS detectors and fragmentation modes.

Supplementary figure 7. Number of notches affects identification sensitivity.

Supplementary figure 8. Optimization of automatic gain control (AGC) targets and maximum injection time (IT).

Supplementary figure 9. Number of total triggered precursors and the spectra identification rates using IgM digests in standard MS2 or Glyco-SPS-MS3 methods with different NCEs.

Supplementary figure 10. Optimization of NCE settings for Glyco-SPS-MS3.

Supplementary figure 11: The effects on the numbers of glycopeptide identification by searching against various refined protein databases.

Supplementary figure 12. Reduced fucosylation in 2FF-treated DG75 cells revealed by lectin blotting and comparison of identifications from DG75 cells using either MS2 or Glyco-SPS-MS3.

Supplementary figure 13. Glyco-SPS-MS3 determined modestly decreased fucosylation in DG75 cells treated with lower concentrations of 2FF.

Supplementary figure 14. An overview of Pearson correlations between replicates. Each biological replicate contains technical triplicates.

Supplementary figure 15. Heterogeneity of glycosylation detected in DG75 cells.

Supplementary figure 16. STRING interaction network of proteins with differentially regulated glycosylation in 2FF-treated DG75 cells.

Supplementary Tables:

Supplementary Table 1. Optimization of MS parameters in Glyco-SPS-MS3 method.

Supplementary Table 2: Suggested MS settings in Glyco-SPS-MS3 for TMT-labelled glycopeptide analysis.

Supplementary note 1

Selection of quantification methods for intact glycopeptides

Using recent advancements in bioanalytical mass spectrometry[1], researchers have employed a variety of methods for quantitative analysis of intact glycopeptides. The heterogeneity of protein glycosylation, however, often makes the quantification challenging. For instance, label-free methods using MS1 extracted ion currents (XICs) or spectral counts have been applied in glycoproteomics with the advantage of simple workflows and lower cost[2-4], but require sophisticated normalization methods to account for the MS response variations in measurements and the varied ionization efficiency of glycopeptides bearing diverse glycan composition[4]. This approach also suffers from severe missing values in large-scale glycoproteomics due to drastic differences in glycoform abundances, and from low identification rates of less abundant glycopeptides in data-dependent acquisition (DDA) analysis[3]. More recently, data-independent acquisition (DIA) methods have shown the potential to quantitate intact glycopeptides with higher sensitivity and less missing values[5, 6]. However, the lack of universal spectral libraries for N-glycopeptides still hampers the applications of DIA methods in large-scale glycoproteomics.

Metabolic labelling, such as stable isotope labelling by amino acids in cell culture (SILAC)[7], allows to combine different samples right after cell harvest, which minimizes possible quantification errors introduced during sample preparation. However, this method is not applicable to many biological materials, especially to patient tissues, and has a limited number (usually up to three) of conditions to be compared in one measurement. In addition, the fact that heterogeneous glycoforms on one peptide core have closely related masses and do not separate well on the standard C18 chromatography often makes MS1 spectra of intact glycopeptide analysis more complicated. SILAC inherently further increases the MS1 complexity, resulting in interfered SILAC pair determination and XIC extraction. The introduced SILAC pair can also cause over-sequencing of the same glycopeptides and under-sampling in DDA analysis.

Isobaric chemical labelling using TMT or iTRAQ reagents, on the other hand, can apply to all types of samples[8]. It enables sample-multiplexing, reducing overall LC-MS measurement time and variations introduced between replicates. Importantly, by pooling all samples together, it boosts the signal of low abundance species that are otherwise not detectable in any individual sample[9]. TMT labelling itself also increases the ionization efficiencies of peptides or glycans[10]. Since the majority of the glycoforms are present with low stoichiometry, this feature enhances the sensitivity and the depth of site-specific glycoproteomics. However, despite its successful applications in quantitative glycoproteomics[11-14], a systematic optimization of experimental parameters for chemically labelled glycopeptides is still missing (see main text). Also, co-isolation interference that occurred in a standard DDA MS/MS analysis can impair the quantification accuracy of chemically labelled peptides and cause ratio compression[15]. Considering the heterogeneity of glycosylation and the closely related masses of glycopeptides, such interference will strongly impact glycoproteomics. We thus developed Glyco-SPS-MS3 in the SugarQuant pipeline to minimize the interference based on the strategy of multi-notch MS.

Another important factor to interfere with glycopeptide quantification is in-source dissociation (ISD), the loss of terminal glycan moieties in source during LC-MS/MS analysis. ISD is problematic for accurate quantification of glycopeptides, especially for those bearing sialic acid. When a glycopeptide is fragmented and loses glycan moieties in the source, the resulting fragmented glycopeptide and its original "parent" glycopeptide will be detected at the same retention time in the liquid chromatography. Once the chromatography does not separate glycopeptides sharing the same peptide sequence but bearing different glycans apart, the newly formed ISD glycopeptide can result in an overlapped ion chromatogram with its natural, unfragmented isomers. MS1-based quantification, including label-free and SILAC, will be compromised by both the reduced intensity of fragmented glycopeptide and the overlapped ion chromatogram. Isobaric labeling-based quantification, on the other hand, is not affected by the former because glycopeptides from all samples are pooled and should theoretically experience equal effects of ISD. However, the ISD glycopeptide and its natural isomers can be co-selected for MS2 and MS3 analyses, leading to an inaccurate quantification of natural glycopeptides.

Supplementary note 2

Development and optimization of sample preparation for quantitative glycoproteomics

Many glycoproteins of biomedical interest participate in processes like e.g. cell surface recognition, and are thus membrane-associated. Complete solubilization especially of these membrane-associated glycoproteins with the assistance of detergents or chaotropic reagents is a critical and necessary step of sample preparation in glycoproteomics. Sodium dodecyl sulfate (SDS) is commonly used because of its outstanding capacity for recovering membrane proteins from a variety of biological materials[16]. However, SDS diminishes trypsin activity and causes severe ion suppression in MS analysis, necessitating an additional removal step prior to MS analysis. Conventionally, SDS is eliminated via protein precipitation. Many other methods have also been developed for detergent removal[17-19]. Unfortunately, those methods increase the risk of sample loss and make the entire procedure laborious and time-consuming. Alternatively, acid-labile detergents, such as RapiGest (Waters), have been demonstrated to improve solubilizing hydrophobic proteins/peptides and facilitate complete proteolysis[20]. They undergo hydrolysis under acidic conditions and is compatible with MS analysis without the need for any extra clean-up steps. Similarly, urea is also commonly used in proteomics as a substitute for detergents to denature proteins and enhance the solubility. A simple desalting step is sufficient to remove urea from the samples, albeit an extensive dilution prior to proteolysis is often required due to its inhibitory effect on proteases at high concentrations.

The selection of detergents or chaotropic reagents directly affects not only the solubility of proteins but also the duration and complexity of the entire sample preparation procedures. We thus compared workflows using SDS, urea and RapiGest for protein extraction (see **Online Methods**). In our hands, SDS and urea outperformed RapiGest, and allowed more proteins and glycoforms to be identified in the following MS analyses (**Supp. Fig. 1a**), although the RapiGest workflow is more straightforward and faster. Considering that residual urea can negatively influence TMT labelling efficiency, SDS becomes the optimal choice for multiplex quantitative glycoproteomics. However, the conventional approach to remove detergent via protein precipitation is too time-consuming. It also takes extra time and efforts to re-dissolve the pellet. We thus sought to simplify the workflow, and reduce handling time.

Recently, Hughes et al. described single-pot solid-phase enhanced sample preparation (SP3) and demonstrated its capacity for fast and efficient proteome sample preparation[21]. Olsen group further investigated the underlying mechanism and found that protein clean-up occurred irrespective of microparticle surface chemistry but instead via protein aggregation capture (PAC)[22]. We removed SDS by PAC on magnetic beads followed by trypsin digestion, which shortened handling time without the need to resolubilize hard protein pellets. We compared the capabilities of different magnetic beads bearing various surface functional groups to retain protein and found that they all worked properly and resulted in less than 5% difference in protein identifications (**Supp. Fig. 1b**). We further reduced the digestion time from overnight to 4 hours and showed no significant decrease in protein identifications (up to 5.3%). Instead, we identified up to 9.1% and 4.1% more glycoforms and glycosites, respectively.

To reduce the cost of TMT reagents, we optimized the ratio of peptides to TMT labeling reagents. In this study, we labeled the peptides in a TMT-to-peptide ratio (wt/wt) of 2:1, a four-fold reduction of TMT reagent than recommended by the vendor (800 μg TMT to 100 μg peptides, 8:1). The labeling efficiency is above 99%. For every biological replicate of the fucosylation-inhibited samples, we labeled 400 μg peptides in each condition using only one set of TMT reagents.

We also introduced basic reverse-phase chromatography (bRP) to pre-fractionate the ZIC-HILIC enriched glycopeptides, and showed its advantages to identify 53% more glycopeptides as compared to repetitive injections (**Supp. Fig. 1c-e**). We further optimized chromatographic settings specifically for TMT-labelled glycopeptides to enhance sensitivity in LC-MS/MS. In summary, the optimized workflow involves SDS-assisted protein extraction, PAC clean-up and proteolysis, TMT labelling, ZIC-HILIC glycopeptide enrichment, and bRP pre-fractionation. The whole workflow can be finished in one day, a three-fold improvement in processing time as compared with a conventional protein precipitation method (**Supp. Fig. 1g)**.

Supplementary note 3

Development of Glyco-SPS-MS3 for confident glycopeptide identification and accurate quantification

Confident glycopeptide identification using tandem MS relies on the match of a comprehensive series of both glycan and peptide fragments from the acquired spectrum. A myriad of different fragmentation strategies has been developed for this purpose[23]. Among others, stepped collision energy HCD (sNCE-HCD) and AI-ETD have recently proved their advantages to achieve large-scale identification of intact glycopeptide in complex samples using rather simple MS acquisition methods and data process workflows[24, 25]. However, none of those methods have been applied to chemically labelled glycopeptides e.g. for multiplexed quantification. Our results suggested the necessity to optimize CE settings specifically for TMT-labelled glycopeptide (see main text). A multi-stage fragmentation method would allow to cover a broader range of CEs for different fragment ion series, including glycan Y ions, peptide b-/y-ions, and TMT reporter ions. Indeed, characterization of glycopeptides using MS3-based methods has shown welcoming advantages[26-28]. For example, by looking for and specifically selecting the Y1 ion (peptide core carrying a single HexNAc) for further fragmentation, additional peptide b-/y-ions could be detected in MS3 to support the peptide sequence identification[29]. However, this requires prior knowledge of the targeted peptides to select for MS3 and sufficient parent ion intensity to generate useful information from the MS3 experiment, thus limiting its throughput and sensitivity. The lack of software tools to automatically interpret and combine information from different fragmentation stages further limits the applicability of MS3 approaches for large scale analysis. To overcome the sensitivity issue of MS3 detection, Gygi and his coworkers developed synchronous (or multi-notch) precursor selection (SPS)[30, 31] to simultaneously select a pre-defined number of MS2 fragments (named notches) for high NCE (65%) fragmentation to produce reporter ions more efficiently. SPS-MS3 has shown to be more accurate in quantification than the standard MS2 approaches because of reduced interference from co-isolated ions.

In this work, we developed Glyco-SPS-MS3 to combine the advantages of multi-stage fragmentation and multi-notch selection for both confident identification and accurate quantification of chemically labelled intact glycopeptides. Unlike the original SPS-MS3 that utilizes fast ion trap scans and paralleled CID fragmentation for high-speed MS2 peptide sequencing, Glyco-SPS-MS3 uses HCD and Orbitrap detection in both MS2 and MS3. Our results show that HCD fragmentation followed by high-resolution, high-accuracy Orbitrap detection provided more high-scored N-glycopeptide identifications from IgM digests **(Supp. Fig. 6)**. Glyco-SPS-MS3 applies different HCD NCEs for MS2 and MS3 fragmentation, resulting in the production of complementary sets of fragments. High-resolution and high-accuracy Orbitrap detection of both MS2 and MS3 fragments reduced mis-identifications. Multi-notch selection not only enhanced the detection sensitivity of both reporter ions and peptide b-/y-ions, but also decreased co-isolation interference. We fine-tuned several key MS instrument parameters as detailed below (**Supplementary Table 1)**. To broaden the applicability, we optimized the settings for both Fusion and Lumos tribrid instruments (**Supplementary Table 2**).

MS acquisition cycle

Original SPS-MS3 fragments isolated precursors with collision-induced dissociation (CID) followed by ion trap MS2 detection. It then selects multiple MS2 fragments for further HCD fragmentation with NCE 65 in the ion-routing multipole (IRM) and sends all resulting ions for Orbitrap MS3 detection. The MS2 and MS3 scans are parallelized to reduce the overall duty cycle. However, we require HCD in our Glyco-SPS-MS3 to generate more Y<5 ions in MS2. In addition, high resolution and high accuracy Orbitrap scans are required to reduce mis-identifications of glycopeptides. Therefore, the entire operation cycle of our Glyco-SPS-MS3 works as follows:

A Glyco-SPS-MS3 cycle starts with an MS1 survey scan detected by the Orbitrap. Top intense ions fulfilled our criteria are selected in the quadrupole, fragmented with HCD using lower NCE, and sent to the Orbitrap for MS2 detection. Among the MS2 fragments, the top ten ions in the *m/z* range of 700-2000 are co-selected using SPS technique in the ion trap, sent back to IRM for HCD fragmentation with higher NCE, and then detected in the Orbitrap.

Because of the use of the Orbitrap for both MS2 and MS3 detection, the operation of Glyco-SPS-MS3 cannot be parallelized. It thus resulted in longer cycle time. We nevertheless deem it worthwhile because of the gained advantages in both identification quality and quantitation accuracy.

Collision Energy

We used 12 TMT-labelled IgM glycopeptides to evaluate the fragmentation patterns under various NCEs, and it suggested the need to apply a wider range of NCE to cover all wanted fragment ions (**Fig. 2a and Supp. Fig. 3**). Stepped NCE helped to generate more glycan Y ions and peptide b-/y-ions, but the resulting reporter ions were still with lower intensities (**Fig. 2c**). Possibly the more labile glycosidic bonds broke first when colliding with gas molecules and took most of the energy that limited the generation of reporter ions[13]. We thus thought that a second-stage fragmentation of the Y ions that bore smaller glycans would result in higher reporter signals, similar to the previous MS3 methods for better detection of peptide b-/y-ions. Instead of choosing only the Y1 ions, we employed the SPS technique to simultaneously select and fragment multiple Y ions for improved sensitivity. We fragmented the TMT-labelled glycopeptide precursor with NCE 25-30 that preferred the production of glycan Y ions and selected only the MS2 fragments in the *m/z* region of 700-2000 for MS3 to exclude intense oxonium ions. We evaluated multiple NCE combinations in the Glyco-SPS-MS3 (**Supp. Fig. 10)** and found that NCE 25 at MS2 plus NCE 35-40 at MS3 indeed resulted in better glycopeptide identification. The resulting total score, peptide score, and glycan score obtained from Glyco-SPS-MS3 were all higher than that from a standard MS2 method.

Ion target and injection time

pGlyco 2.0 considers both glycan and peptide fragments in the scoring algorithm with FDR control so that it demands high spectrum quality to identify a glycopeptide. Consistent with previous reports[24, 26], our results showed that higher AGC targets helped to identify more glycopeptides with either pGlyco or Byonic search engines (**Supp. Fig. 8**). We reasoned that higher AGC benefited the identification of low abundance and/or low ionization efficiency glycopeptides. However, it required longer ion accumulation time (or ion injection time, IT) to reach the desired ion amounts, resulting in prolonged cycle time during MS/MS analysis. We used 100 ms of IT to analyze IgM digests and found that only 81%, 70%, and 5% of the spectra reached the pre-defined AGC targets of $5e^4$, $1e^5$, and $5e^5$, respectively. Prolonging the IT to 250 and 500 ms allowed 29% and 68% spectra, respectively, to reach the AGC of $5e^5$. However, using 500 ms IT for both MS2 and MS3 in our Glyco-SPS-MS3 would make the duty cycle too long to couple with LC separation. We thus decide to use the maximum IT of 500 ms for a precursor and allocate it to MS2 and MS3 scans variously. Interestingly, longer IT in MS3, or shorter IT in MS2, led to increased peptide score along with slightly decreased glycan score. This observation is in agreement with our suggestions that low-NCE MS2 provided more glycan Y ions, while high-NCE MS3 generated more peptide b/y ions. Of note, we did not observe any deviated mass accuracy caused by the space charging effect[32] with these settings.

We compared the required cycle time of our Glyco-SPS-MS3 with previously published acquisition methods including HCD triggered AI ETD, ETD and EThcD methods by Rieley *et al*[25]. The MS1 acquisition parameters were essentially the same, requiring 120,000 resolution at 200 m/z and maximum injection time of 50 ms and automatic gain control set to 400,000 (Rieley *et al.*) or 500,000 (SugarQuant). The cycle time was automatically controlled by the machine and set to 3 s. Therefore, the main factor that limits acquisition speed and affects the overall cycle time is the total maximum injection time allowed for the MS2-MS3 or HCD-triggered (AI)ET(hc)D scan pairs. Riley et al allocated 460 ms for an HCD/triggered (AI)ET(hc)D scan pair, including 60 ms for a survey HCD scan and 400 ms for a triggered (AI)ET(hc)D scan. In our suggested Glyco-SPS-MS3 settings, we allocated 500 ms for an MS2/SPS MS3 scan pair in total, with 150 ms being used by an MS2 scan and 350 ms being allocated for an SPS MS3 scan. This resulted in an 8% increase in the total time required for an MS2/SPS MS3 scan pair as compared with the triggered ETD methods. The required ion reaction time for (AI-)ET(hc)D, however, is often longer than that for HCD, which makes the difference in overall cycle time between the methods negligible. Importantly, SugarQuant utilizes both MS2 and SPS-MS3 scans and thus brings in additional advantages for reliable glycopeptide identification as well as accurate quantification.

Supplementary note 4

Development and usage of GlycoBinder

GlycoBinder is written in R and is available on GitHub (https://github.com/IvanSilbern/GlycoBinder). It allows streamlined data processing of multiplexed glycopeptide quantitative mass spectrometry data. It relies on the usage of external tools (see below) that are not distributed with the script and have to be requested and installed separately.

External tools

1. RawTools, version 2.0.2 [https://github.com/kevinkovalchik/RawTools][33]
2. msconvert (ProteoWizard), version 3.0.19262 (0a01c36ac) [https://github.com/ProteoWizard/pwiz][34]
3. pParse, version 2.0.8 [http://pfind.net/software/pParse/index.html][35]
4. pGlyco, version 2.2.0 [http://pfind.net/software/pGlyco/index.html][24]

GlycoBinder does not provide those tools and a user needs to request and install the tools by himself prior to working with GlycoBinder. To our knowledge, all tools are freely available upon request.

Setting up the processing environment

GlycoBinder was developed and tested on machines running on 64-bit platforms under Windows 10. It requires an R programming language (versions 3.5.0 or above) to be installed on your machine including *data.table*, *dplyr*, *future.apply*, and *stringr* packages. In case those packages are not installed, GlycoBinder will make an attempt to install them. Note the location of "Rscript.exe" file, which is needed to run R scripts in command line (commonly in C:/Program Files/R/R-3.5.0/bin/x64/)

External tools should be installed and added to the system path of the machine. This allows for calling the program without specifying an exact path to it. To do so, open windows menu and search for "Edit environment variables for your account". Under "User Variables" select "PATH" and click the "Edit" button (make sure you are changing the "PATH" variable for a user account you will be later working with). Select "New" and then "Browse". Navigate to the directory where the executable of the tool is located (e.g. "C:\Program Files\RawTools-2.0.2\"). Repeat the same procedure for all tools. We also suggest to add the file path to the folder containing "Rscript.exe" file. After the environmental variables are configured, please check if the programs can be accessed from the command line directly. For this, open the command line and type one by one: RScript, rawtools, msconvert, pparse, pglyco. Hit Enter after each command. Make sure that the system can find each tool and returns help information to the console. A tutorial on how to configure the different environmental variables can be found here: https://github.com/kevinkovalchik/RawTools/wiki/Download-and-prepare-RawTools-for-Windows

Depending on the number of raw files and their size, GlycoBinder might require a large amount of RAM to process the data. Per default, it will use (the number of available processors – 2) threads on your machine for processing the data (this number might vary for external tools). We recommend to reserve at least 1GB of free RAM per running process (e.g. for a machine with 8 cores, one should aim for at least 6 GB of free RAM space). If you would like to restrict the number of processors used by GlycoBinder, please, consult the following section regarding additional parameters to the script.

Processing steps

GlycoBinder is designed for processing .raw files acquired on Thermo Fisher Orbitrap Tribrid instruments. It combines MS2 scans with their dependent MS3 scans. It also extracts reporter ion intensities for subsequent quantification.

In brief, GlycoBinder processes the .raw files using the following steps:

1. RawTools extracts reporter ion intensities from Thermo .raw files and assigns MS3 scans to their parent MS2 scans.
2. msconvert transforms .raw files into .mgf file format and centroids data by applying a vendor-specific peak picking algorithm. Both MS2 and MS3 scans are preserved in the .mgf file.
3. pParse recalibrates the monoisotopic peaks of precursors and outputs an .mgf file containing MS2 scans.
4. GlycoBinder combines ion intensities of matching MS2 and MS3 spectra as reported by RawTools. MS2 and MS3 spectra are extracted from the msconvert-produced .mgf file, and merged based on the specified mass tolerance window. GlycoBinder replaces MS2 spectra in the pParse output by the combined MS2/MS3 spectra. The modified pParse output file is used as input for pGlyco 2.

5. pGlyco 2 uses the combined MS2/MS3 spectra to search for peptides and associated glycans. After the first pGlyco 2-search is finished, results are filtered based on a specified FDR cutoff.
6. Optionally, a second pGlyco 2-search is performed with a smaller protein database. For this, only glycoproteins identified in the first round of pGlyco 2-search passing an FDR threshold are retained in the protein sequence database and used for the second round of glycopeptide search.
7. GlycoBinder combines the resulting GPSMs with the corresponding reporter ion intensities extracted from the same spectra by RawTools. Based on the combined pGlyco 2 and RawTools output, GlycoBinder organizes quantitative results at different levels: at the levels of glycosylated peptides, glycoforms, glycosites, and glycans. A separate data table is reported for each level that contains unique identifiers of the data entries, cross-references to other levels, quantification information in the form of the summed reporter ion intensities and necessary metadata. By re-organizing the combined pGlyco 2 and RawTools output, Glycobinder allows to directly address changes happening at the level of glycopeptides, glycoforms, glycosites or glycans since the quantitative information is conveniently structured at each level.

Use of GlycoBinder

To execute GlycoBinder, follow these steps:

1. Prepare a working directory containing .raw files to be processed and .fasta file containing amino acid sequences of proteins.
2. Open the command line
3. Specify the path to the Rscript.exe (or just "Rscript.exe" if the file path is set in environmental variables)
4. Specify the path to the GlycoBinder.R file
5. Specify the path to the working directory using --wd flag
6. Specify peptide labelling reagent after --reporter_ion flag (values supported by RawTools are allowed: TMT0, TMT2, TMT6, TMT10, TMT11, iTRAQ4, iTRAQ8), e.g. --reporter_ion TMT6
7. Specify additional arguments (s. below)

Supposing that .raw files, the .fasta file, and the *GlycoBinder.R* script are located in *C:/data*, and peptides were labelled using TMT6plex reagents, the minimum required input would look like:

C:/data>Rscript.exe "GlycoBinder.R" --wd "C:/data" --reporter_ion TMT6

Additional parameters to GlycoBinder

Following parameters modify default GlycoBinder behavior if added as command line arguments:

1. --verbose
   Force GlycoBinder to be more chatty.
2. --tol_unit
   Specify tolerance unit used for matching ions from corresponding MS2 and MS3 spectra. Supported values are ppm and Th, e.g. --tol_unit ppm (default).
3. --match_tol
   Specify tolerance for matching ions from corresponding MS2 and MS3 spectra. Integer numbers are supported, e.g. --match_tol 1 (default). Default tolerance widow for ion matching is 1 ppm. It means, if two ions in the matching spectra have an absolute mass difference smaller than 1 ppm, those peptides will be considered the same and their intensities will be summed.
4. --pglyco_fdr_threshold
   Specify total FDR cutoff for pGlyco 2 search results, e.g. --pglyco_fdr_threshold 0.02 (default) sets maximum total FDR to 2%.
5. --no_second_search
   Prevent GlycoBinder from running second pGlyco 2 search on reduced data base.

6. --report_intermediate_results
   Forces GlycoBinder to keep intermediate files (after pGlyco 2 search).
7. --nr_threads
   Specify number of available processors for GlycoBinder processing. It can take values between 1 and the number of available processors - 2 (default).
8. --seq_wind_size
   The parameter specifies the number of amino acids around the modification site. It is applied to extract sequence window around modification site from protein sequences. Sequence windows are needed to combine quantitative information on glycoform level. Default paramter is 7, e.g. --seq_wind_size 7. Seven amino acid before the modified site and seven amino acids after the modified site will be extracted, resulting in the 15 amino acids long sequence window.

Default parameters for external tools

Per default, external tools are used with parameters listed below. The majority of the parameter cannot be changed through GlycoBinder. However, one can execute those tools outside of GlycoBinder using a different parameter set and then supply the output files into the respective folder within the GlycoBinder working directory (specified after --wd flag while running the script). In this case, GlycoBinder skips execution of a respective tool.

1. **RawTools**
   rawtools -parse -d [input directory] -out [output directory] -q -r [reporter ions type] -R –u

   RawTools output one _Matrix.txt file per .raw file. Output file names are created by appending _Matrix.txt to the .raw file name including extension (example: "raw_file.raw" becomes "raw_file.raw_Matrix.txt"). RawTools output files are located in ./rawtools_output folder within the specified working directory. One can process raw files externally and then copy the resulting _Matrix.txt files into the ./rawtools_output folder. If every .raw file has a corresponding _Matrix.txt file, GlycoBinder will skip RawTools processing.

2. **msconvert**
   msconvert [file] --outdir [output directory] --mgf –ignoreUnknownInstrumentError --singleThreaded --filter "peakPicking vendor" --filter "defaultArrayLength 1-" --filter "titleMaker <RunId>.<ScanNumber>.<ScanNumber>.<ChargeState>"

   Similar to RawTools, msconvert outputs one .mgf file per .raw file in the GlycoBinder working directory. Output file names are equal to the input file name with .raw extension substituted by by .mgf. msconvert output files are located in ./msconvert_ouput folder within GlycoBinder working directory. If GlycoBinder can locate all .mgf files in the ./msconvert_output folder, the msconvert processing step is skipped. For correct processing of .mgf files generated by msconvert, each scan within an .mgf file should contain a line starting with "TITLE=" and containing a scan number flanked by dots, e.g. ".355.".

3. **pParse**
   pParse.exe -D [file] -O [output directory] -p, 0

   pParse output files are located in ./pparse_output folder and named as original .raw files with .raw file extension substituted by _[Type of Detector, e.g. CDFT or ITFT].mgf. Similarly, GlycoBinder processing is skipped if all output files are found within the ./pparse_output folder. After merging of MS2 and MS3 spectra, MS2 spectra within pParse output files are substituted by the combined MS2/MS3 spectra. The modified pParse output files are renamed to [base_raw_file_name]_pParse_mod.mgf files and saved in the same ./pparse_output folder. If all _pParse_mod.mgf are found in the ./pparse_output folder, pParse processing and merging of the MS2 and MS3 spectra are skipped.

4. **pGlyco 2**

pGlycodb.exe [pglyco configuration file] && pGlycoFDR.exe -p [pglyco configuration file] -r [output file name] && pGlycoProInfer.exe

pGlyco 2 workflow consist of three programs, pGlycodb.exe, pGlycoFDR.exe, and pGlycoProInfer.exe that are executed one after another and rely upon configuration file that should be created before the first program has been called. If GlycoBinder does not find any file with a name pGlyco_task.pglyco in the working directory, it will create a configuration file with default parameters. One can create its own configuration file, e.g. using graphic user interface of pGlyco 2, name it as pGlyco_task.pglyco and then copy it to the working directory of GlycoBinder. In this case, pGlyco 2 will utilize the existing parameter file for glycopeptide search. Following parameters are used per default and can be changed when supplying a GUI-created pGlyco_task.pglyco file to the GlycoBinder working directory:

- enzyme=Trypsin_KR-C
- max_miss_cleave=2
- max_peptide_len=40
- min_peptide_len=6
- max_peptide_weight=4000
- min_peptide_weight=600
- [modification]
- fix_total=3
- fix1=Carbamidomethyl[C]
- fix2=TMT6plex[K]
- fix3=TMT6plex[AnyN-term]
- max_var_modify_num=3
- var_total=1
- var1=Oxidation[M]
- [search]
- search_precursor_tolerance=10
- search_precursor_tolerance_type=ppm
- search_fragment_tolerance=20
- search_fragment_tolerance_type=ppm

When using a protease different from trypsin, it is important to assure that: a) the protease is configured in "./pGlyco/2.2.1/bin/enzyme.ini" file and b) pGlyco 2 configuration file is located in the working directory of GlycoBinder. Change "enzyme=Trypsin_KR-C" to "enzyme=[Name_of_enzyme]" in the configuration file and save it under "pGlyco_task.pglyco". Similar procedure applies when configuring pGlyco 2 search with a different set of modifications. Other parameters in the configuration file will be overwritten irrespectively of the origin of the configuration file. The same parameter file will be used in the second pGlyco 2 search.

The output file is pGlycoDB-GP-FDR-Pro.txt for the first pGlyco 2 search and pGlycoDB-GP-FDR-Pro2.txt for the second search, respectively. Both files are located in the ./pglyco_output folder. If the file pGlycoDB-GP-FDR-Pro.txt exists (or pGlycoDB-GP-FDR-Pro2.txt exists and --no_second_search flag was not used), GlycoBinder will skip the first (or first and second) pGlyco 2 search, respectively.

Special case: MS2 data

After processing with RawTools, files that were identified as not containing MS3 scans will not be subjected to msconvert processing. The MS2/MS3 spectra merging step is skipped as well. After pParse processing, original pParse output files are renamed to _pParse_mod.mgf files for consistency and used as input for pGlyco directly.

Merging of MS2/MS3 spectra

GlycoBinder combines MS2 and MS3 spectra based on MS2 and MS3 spectra scan number pairs in the RawTools output files (*MS2ScanNumber* and *MS3ScanNumber* columns within _Matrix.txt file). First, ions from MS2/MS3 scan pairs are roughly matched using 1 Th tolerance window. Initially matching ions are then tested to satisfy the specified tolerance window (1 ppm per default, it can be changed by specifying --tol_unit and --match_tol arguments). If several ions matches the same ion, the ions with the minimal absolute mass difference are considered as a matching ion pair. Intensities of matched ions are summed. Remaining MS3 ions that do not have matching MS2 ions are simply added to the MS2 spectra. pParse .mgf file then will output merged MS2/MS3 spectra. GlycoBinder matches spectra in the pParse output file to the merged MS2/MS3 spectra based on the scan number. While scan number is unique for merged MS2/MS3 spectra, several spectra in the pParse output can refer to the same scan number. For all of them, the spectrum will be substituted by the respective merged MS2/MS3 spectrum. Spectra that do no share scan number with merged MS2/MS3 spectra will be kept unchanged.

GlycoBinder output

GlycoBinder stores the output of the external tools in separate folders: rawtools_output, msconvert_output, pparse_output, and pglyco_output. After the processing, files located in rawtools_output, msconvert_output, and pparase_output folders can be removed. However, keeping those files would allow for faster data re-processing, as GlycoBinder can skip certain processing steps (e.g. .mgf generation by msconvert) if the output files generated by the external tool is already present. Differently, the pglyco_output folder contains not only the pGlyco 2 output files from the first and the (optional) second glycopeptide search (pGlycoDB-GP-FDR-Pro.txt and pGlycoDB-GP-FDR-Pro2.txt, respectively), but it also contains result data tables created by GlycoBinder:

1. pglyco_quant_results.txt

The table represents a combination of pGlyco 2 output (pGlycoDB-GP-FDR-Pro.txt or pGlycoDB-GP-FDR-Pro2.txt) and RawTools output files (_Matrix.txt files). Quantitative information from RawTools output is merged with pGlyco 2 output file based on the .raw file name and MS2 scan number. Each row represents an identified spectrum (pGlyco 2) with extracted reporter ion intensities (by RawTools). Column names from pGlyco 2 and RawTools are preserved and their descriptions can be found in the documentation for pGlyco 2 and RawTools.

2. pGlyco_Scans.txt

The table is pglyco_quant_results.txt table filtered in the accordance with the total FDR cutoff (lesser than 2% FDR per default, the default cutoff can be changed when specifying --pglyco_fdr_threshold parameter). Column *id* is added for cross-reference with following tables.

3. pGlyco_modified_peptides.txt

The table is based on pGlyco_Scans.txt. Each row contains information about a modified peptide (glycopeptide) – a peptide with a specific glycan composition. Scans belonging to the same modified peptide are combined and their reporter ion intensities are summed. Additional variable modifications of the peptide are not taken into account. Accordingly, reporter ion intensities are combined if the glycopeptide is identified in different .raw files. Glycopeptides carrying a missed cleavage site are considered as individual glycopeptides and not merged with their fully cleaved counter-parts. Precursor information from different scans is concatenated using the default pGlyco 2 separator ("/"). *pGlyco_ids* column refers to *id* column in the pGlyco_Scans.txt table and can be used to identify original scans contributed to a particular glycopeptide. *Leading_Protein* and *Leading_ProSite* columns report a representative protein from the protein group and corresponding glycosylated site based on the selection criteria discussed below.

4. pGlyco_glycoforms.txt

The table is based on pGlyco_modified_peptides.txt. Each row contains information about a glycoform – a glycan attached to a particular site on the protein sequence. Amino acids surrounding the glycosylated site form a sequence window. Sequence window in combination with glycan composition is used to distinguish different glycoforms. Sequence windows are first extracted from the amino acid sequences of corresponding proteins. Per default, +/-7

amino acids are extracted around the modification site (the number can be changed if specifying --*seq_wind_size* parameter). Glycopeptides that share the same modification site are grouped together and form a peptide group. Sequence windows are extracted from proteins that contain those peptides and ranked based on the number of peptides each sequence window can explain. Ties are broken by protein ranking (see description below). Peptides shared among several sequence windows are assigned to the sequence window that encompasses the majority of the peptides within the peptide group. If there are peptides that cannot be explained by the leading sequence window, those peptides are distributed between other sequence windows accordingly. Intensities of glycopeptides sharing same sequence window (*seq_win*) and glycan composition (*Glycan(H,N,A,G,F)*) are summed. Descriptive information is concatenated using ";" as a separator. *modpept_ids* refers to the *id* column in the pGlyco_modified_peptides.txt table. It contains the ids of glycopeptides that contributed to a particular glycoform.

5. pGlyco_glycosites.txt

The table is based on pGlyco_modified_peptides.txt table. Each row contains information about a glycosite – a glycosylated site on a protein sequence irrespective of particular glycan composition. Since there might be certain ambiguity in assignment of peptides to proteins, sequence windows are used to define the glycosite in practice. Accordingly, the table contains *seq_win* column with sequence window information, *modpept_id* column that refers to *id* column in the pGlyco_modified_peptides.txt. Intensities of glycopeptides sharing the same sequence window are summed. Descriptive information is concatenated using ";" as a separator. *Leading_Protein* and *Leading_ProSite* are selected according to protein rank. Proteins are ranked based on the number of unique peptides (highest priority), number of all peptides, number of glycoforms assigned to the protein, whether it is a Swiss-Prot entry, and whether it is a canonical sequence or is an isoform (lowest priority). Proteins that have greater numbers of unique peptides/total peptides/glycoforms, are annotated in Swiss-Prot and represent a canonical sequence, receive a higher rank. The highest rank is 1. The rank is unique and ties, if occur, are broken by alphabetic order.

6. pGlyco_glycans.txt

The table is based on pGlyco_modified_peptides.txt table. Each row contains information about a unique glycan composition identified in the data set. The information about the peptide sequence is not taken into account. The inten information is combined based on glycan composition only (*Glycan(H,N,A,G,F)* column). *modpept_id* column refers to *id* column in the pGlyco_modified_peptides.txt and can be used to link glycopeptides carrying a particular glycan composition. Columns *pGlyco_ids*, *Scan*, *Leading_Protein*, *Leading_ProSite* are concatenations of respective columns in pGlyco_modified_peptides.txt using ";" as a separator.

Special case: use of another search engine

Currently, pGlyco 2.0 is the only search engine supported by the GlycoBinder workflow. However, GlycoBinder reports merged MS2/MS3 spectra in mgf format that are located in ./pparse_output folder and marked with the "_mod.mgf" suffix. These mgf files can be used with any other search engine compatible with the mgf format. The search engine output then has to be integrated with the quantitative data from RawTools output ("_Matrix.txt" files in ./rawtools_output folder) manually. Scan numbers and raw file names can be used to integrate qualitative and quantitative information, respectively.

Demonstration data set

As a test data set, we provide an IgM_TMT0.raw file. It is a tryptic digest of a purified IgM sample labeled with TMT0 reagent. The file is located in the "demo" folder together with a Human_IgM.FASTA file containing amino acid sequences of the two human proteins, IgM and IgJ, respecitvely. To test the performance of the GlycoBinder, download the contents of the "demo" folder (e.g. into *C:/data/Glycobinder/demo*), copy the current version of GlycoBinder into it and execute in the command line using following parameters:
C:/data/Glycobinder/demo>Rscript.exe "GlycoBinder.R" --wd "C:/data/Glycobinder/demo" --reporter_ion TMT0 --no_second_search

If you download files from GitHub using git bash, please first install git lfs (https://git-lfs.github.com/) that is aimed at handling large files (e.g. the example raw file). If you use web interface for downloading ("download ZIP"), you will download a placeholder for IgM_TMT0.raw file. To download the actual file, find it in the GitHub repository and click on "View raw". Save the file in your local "demo" folder.

The execution takes around 5 min on a desktop computer running Windows 10 and equipped with Intel Core i7-6700 CPU (64 bit) and 32 Gb of RAM. Beware that the execution time will scale up with the complexity of the data set provided.

GlycoBinder output is located within pglyco_output folder. There are 67 glycoforms (pGlyco_glycoforms.txt), 45 unique glycan compositions (pGlyco_glycans.txt) and 4 glycosylation sites (pGlyco_glycosites.txt) identified in the data.

Please see https://github.com/IvanSilbern/GlycoBinder for further details.

Supplementary Figures

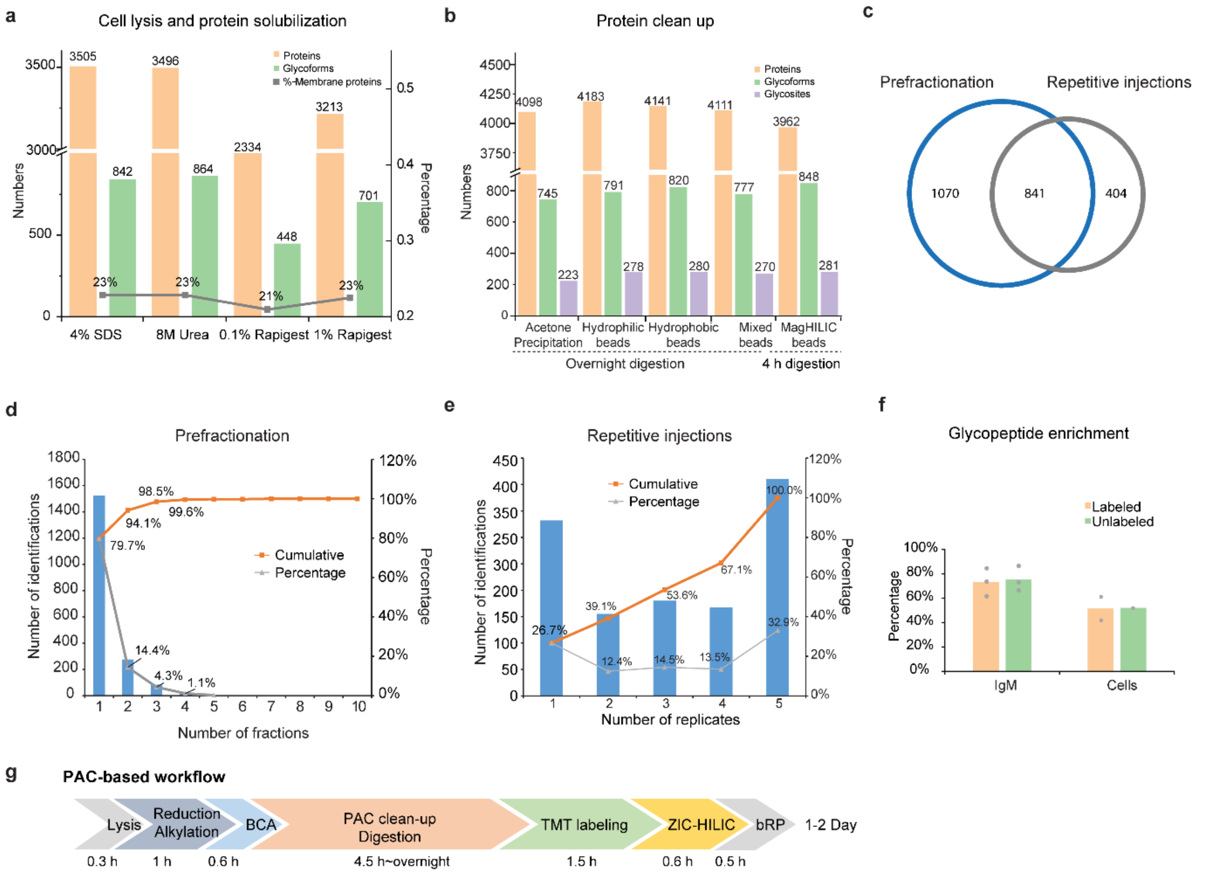

Supplementary figure 1. Optimization of experimental parameters for sample preparation of TMT-labelled glycopeptides. (a) Four different lysis buffers were used for protein extraction and solubilization from DG75 cells. Using the sample preparation methods described in Online Methods, the resulting (glyco)peptides from cell lysate before or after ZIC-HILIC enrichment were analyzed by LC-MS/MS with two replicates. The number of identified proteins and glycoforms were shown in orange and green, respectively. Among the identified proteins, percentages of membrane-associated proteins were also shown. (b) Comparison of the conventional acetone precipitation with PAC methods using various types of beads for protein clean-up (extracted from DG75 lysate). "Hydrophilic beads" are Sera-Mag SpeedBeads with a hydrophilic surface (GE Healthcare, cat.no. 45152101010250, Magnetic Carboxylate Modified), "Hydrophobic beads" are Sera-Mag SpeedBeads with a hydrophobic surface (GE Healthcare, cat.no. 65152105050250, Magnetic Carboxylate Modified), "Mixed beads" is a mixture of hydrophilic and hydrophobic beads at a mass ratio of 1:1, and "MagHILIC beads" are MagReSyn HILIC (ReSynBio, cat.no. MR-HLC005) with a mixed-mode functional surface. The numbers of identified protein, glycoforms, and glycosites were shown in orange, green, and purple, respectively. Note that the MagHILIC used 4-hour digestion while others were digested overnight. Two replicates were included for all experiments. (c) Comparison of the glycopeptide identifications (enriched from Daudi cells) through bRP prefractionation or up to five repetitive injections using a 50 cm column and longer gradient without prefractionation. (d) The distribution of identified glycopeptides in different numbers of fractions. 79.7% of total glycopeptides were identified in one fraction, and 14.4% were in two fractions. (e) The distribution of identified glycopeptides in different numbers of injection replicates. 26.7% were exclusively identified in one of the injections, and 32.9% were identified in all five replicates. (f) Comparison of glycopeptide enrichment specificity between unlabelled (orange) and labelled samples (green) from IgM or DG75 cell samples. We defined the enrichment efficiency by taking the ratio of glycan-oxonium-ion-containing spectra versus all MS2 scans in an LC-MS/MS run. The results included triplicate

and duplicate sample preparations of IgM and cell samples, respectively. (g) The timeline of the optimized PAC-based workflow. Note that the time required for lyophilization is variable in a sample dependent manner and thus not included here. Source data are provided as a Source Data file.

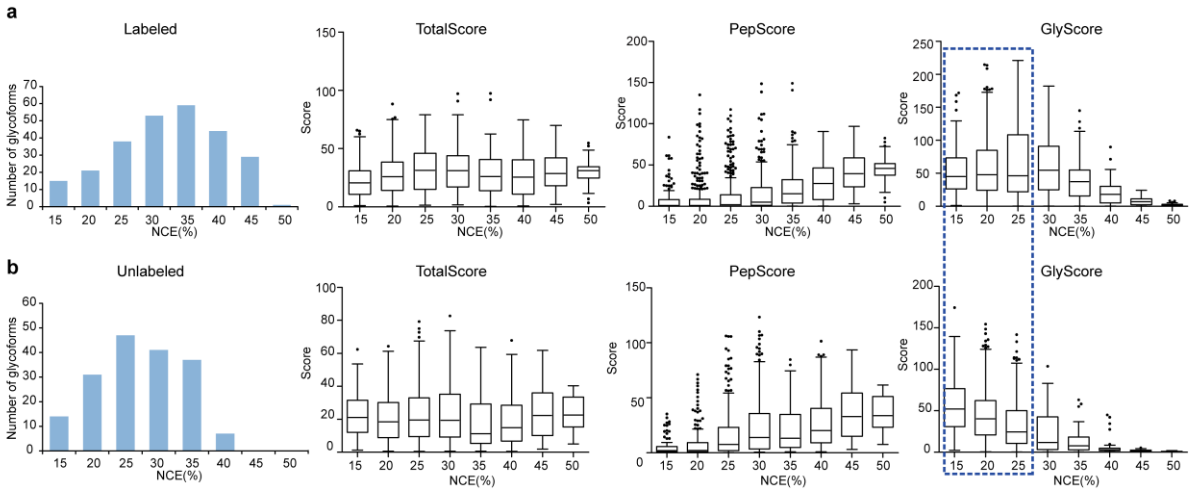

Supplementary figure 2. Effects of TMT-labelling on glycopeptide identification. TMT-labelled (a) or unlabelled (b) IgM glycopeptides were analyzed separately with LC-MS/MS using various HCD-NCEs. The optimal NCEs to achieve the best (from left to right) numbers of identified glycoforms, total scores, peptide scores (PepScore), and glycan scores (GlyScore) in subsequent database search using pGlyco 2 are compared. The blue dashed line highlights the most distinctive score distributions caused by TMT-labelling. The numbers of data points, i.e., numbers of glycopeptides, used in all panel a boxplots are (from left to right): 182, 276, 308, 285, 273, 240, 158, and 59. The numbers of data points used in all panel b boxplots are (from left to right): 114, 231, 245, 184, 127, 108, 66, and 29. Boxplots show the median (centerline), first and third quartiles (box edges) and 1.5 × the interquartile range (whiskers) and outliers. Source data are provided as a Source Data file.

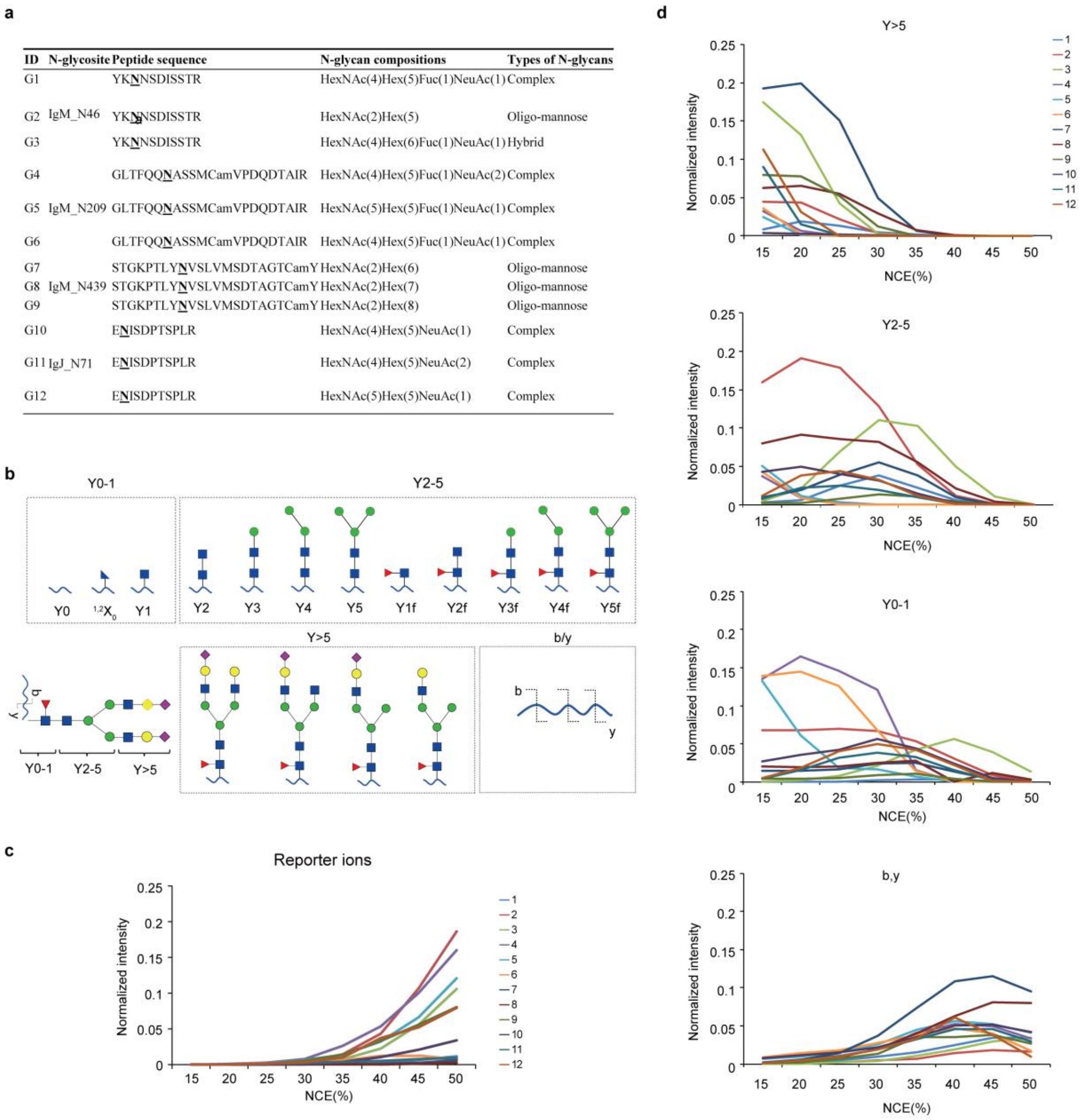

Supplementary figure 3. Fragmentation of TMT labelled N-glycopeptides under different HCD NCEs in LC-MS/MS analyses. (a) Twelve TMT-labelled N-glycopeptides from IgM were selected to monitor their fragmentation patterns under various NCE (see online method). (b) All potential product ions of an N-glycopeptide were classified into four categories based on their nature and the size of attached glycan moiety: Y0-1, intact peptide backbone attached with zero to 1 monosaccharide, including Y0, $^{0,2}X_0$ ions (cross-ring fragmentation) and Y1; Y2-5, intact peptide backbone attached with N-glycan core structure, including Y2, Y3, Y4, Y5 and Y1f, Y2f, Y3f, Y4f and Y5f; Y>5, intact peptide backbone with N-glycans extending from the five-sugar N-glycan core structure; b/y ions, peptide fragments without glycan attached. (c,d) The intensities of reporter ion (c) or fragment ions (d) of TMT-labelled glycopeptides detected under different NCEs in eight consecutive spectra were extracted and normalized to the total ion current of the respective spectra. Fragment ion intensities of different glycopeptides are color-coded as shown on the right. Source data are provided as a Source Data file.

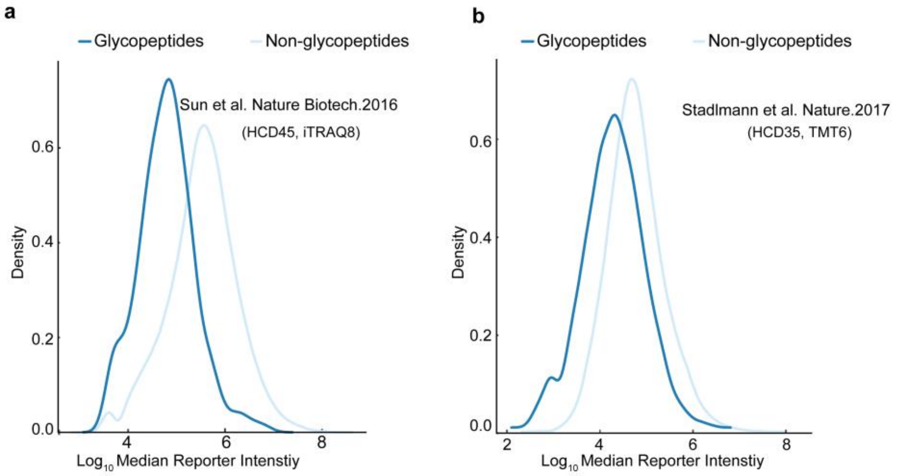

Supplementary figure 4. Kernel distributions of detected reporter ion intensities in glycopeptide or non-glycopeptide from previous publications. The data was retrieved from previous publications[11, 12] as indicated in the figure. We also note the used chemical labelling reagents and applied NCEs in the figure. We log-transformed the reporter intensities and took the median of all reporters on each MS2 spectrum for comparison. Reporter ions from glycopeptides and non-glycopeptides were labelled in dark and light blue, respectively. Source data are provided as a Source Data file.

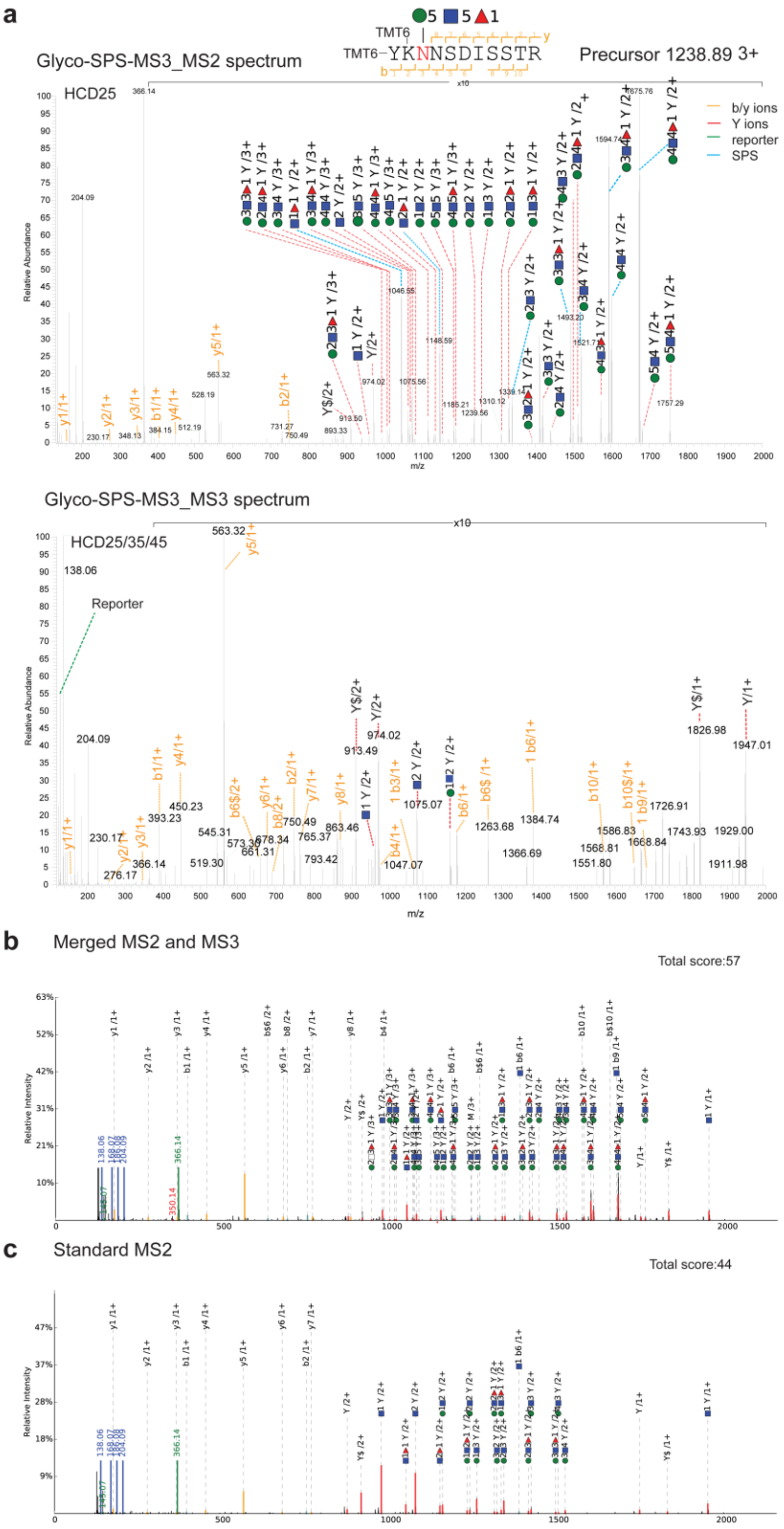

Supplementary figure 5. Representative MS2 and MS3 spectra of a glycopeptide YKNNSDISSTR+Hex5HexNAc5Fuc acquired using standard HCD MS2 or Glyco-SPS-MS3. (a) Annotated MS2 (upper) and MS3 (bottom) spectra of the glycopeptide using Glyco-SPS-MS3. All matched Y ions (red lines), b/y ions (yellow lines), and reporter ions (green ions) are marked. The top ten precursors selected for MS3 are marked with blue lines. Magnified m/z regions of the spectra are highlighted with the magnitude specified. (b) The MS2 and MS3 spectra from (a) were merged and subjected to pGlyco 2 database search. The matched fragment

ions were reported using pLabel, a spectral visualization tool bundled with pGlyco 2. (c) A standard HCD MS2 spectrum from the same selected glycopeptide.

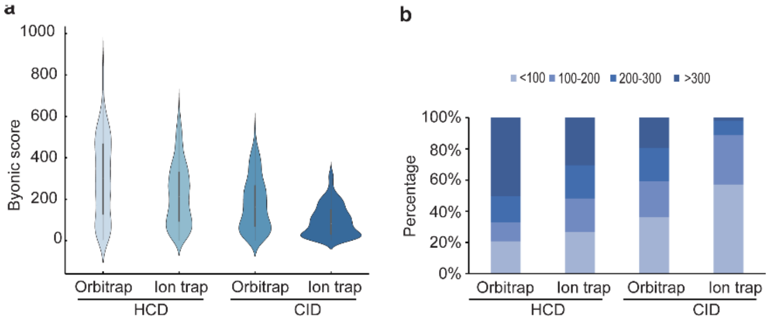

Supplementary figure 6. Selection of MS detectors and fragmentation modes. We examined how different MS detectors (Orbitrap versus ion trap) and fragmentation methods (HCD versus CID) affected IgM glycopeptide identification from triplicate measurements. (a) The distribution of Byonic scores obtained from 4 different combinations (Orbitrap_HCD, Ion trap_HCD, Orbitrap_CID and Ion trap_CID). Numbers of glycopeptides for the violin plots (a) are (from left to right): 659, 1257, 483, 768. Violin plots show the median (white dots), first and third quartiles (box edges) and 1.5 × the interquartile range (whiskers). (b) Stacked bar chart showing the quality of glycopeptide identification using different combination. GPSMs were grouped based on their Byonic scores. A Byonic score of ≥300 was suggested as the cut off for confident identification of glycopeptides[36]. Source data are provided as a Source Data file.

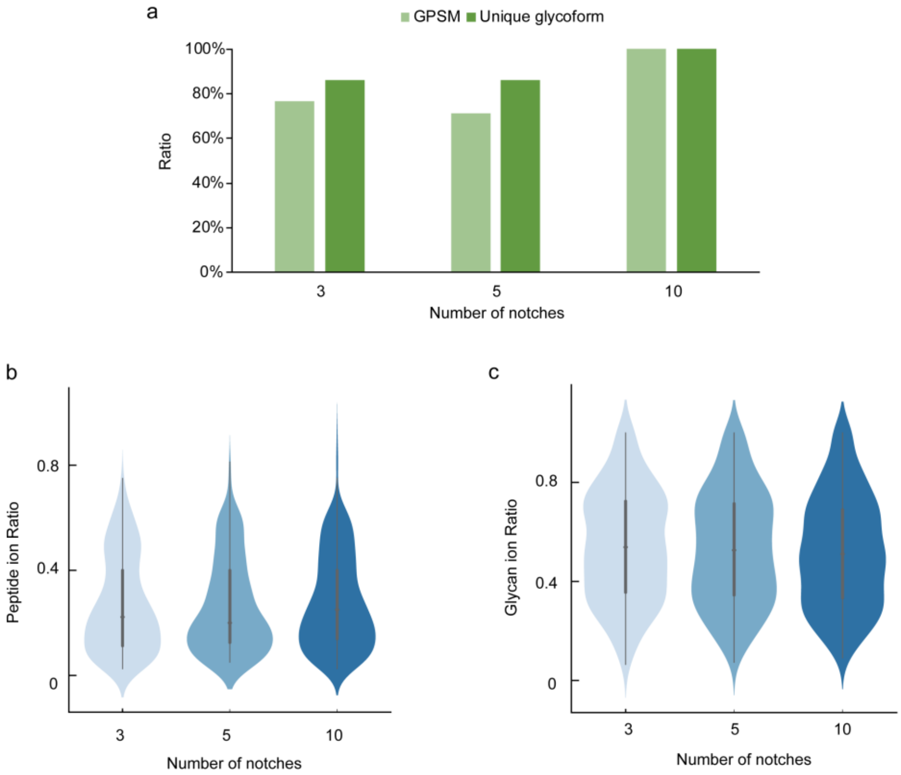

Supplementary figure 7. **Number of notches affects identification sensitivity**. We used various numbers of MS2 fragments to be co-selected for MS3 analysis (number of notches) and examined how the setting affected (a) the numbers of identified GPSMs (light green) or glycoforms (dark green) from IgM, the identifications were normalized to the highest number of GPSM or glycoform; and the overall distributions of peptide ion ratio (b) and glycan ion ratio (c). Numbers of glycopeptides used in both of the violin plots are (from left to right): 380, 439, and 520. Violin plots show the median (black dots), first and third quartiles (box edges) and 1.5 × the interquartile range (whiskers). Source data are provided as a Source Data file.

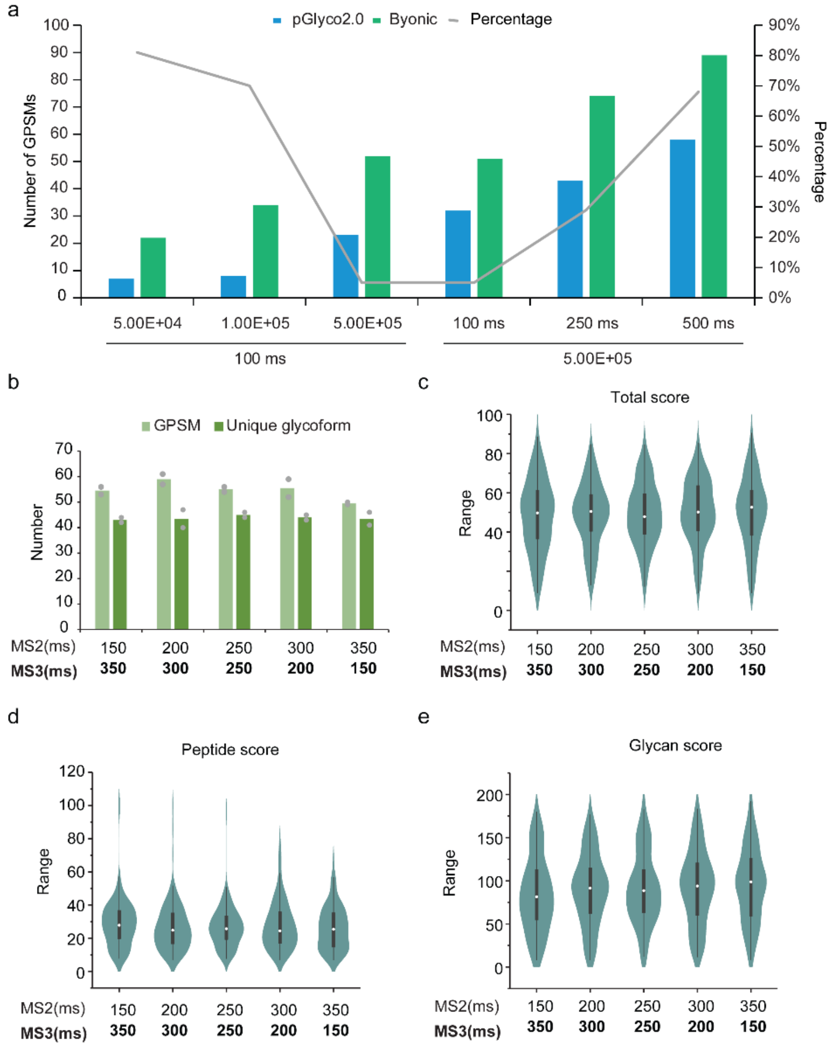

Supplementary figure 8. Optimization of automatic gain control (AGC) targets and maximum injection time (IT). (a) Numbers of GPSMs identified by either pGlyco2.0 (blue) or Byonic (Green) using different settings of AGC targets and maximum injection time in standard MS2 runs. The percentages of MS/MS scans reaching pre-defined AGC were also included. All identification required stringent criteria: pGlyco 2.0: PepScore >7 and GlycScore>8; and Byonic: score >300. (b) Numbers of identified GPSMs (light green) and unique glycoforms (dark green) from IgM digests in Glyco-SPS-MS3 (two independent technical replicates). Used ITs in MS2 or MS3 scans were shown at bottom and the AGC for both MS2 and MS3 are $5e^5$. Distributions of total score (c), peptide score (d), and glycan score (e) were also shown. Numbers of data points used in all violin plots are (from left to right): 109, 118, 110, 111, and 99. Violin plots show the median (white dots), first and third quartiles (box edges) and 1.5 × the interquartile range (whiskers). Source data are provided as a Source Data file.

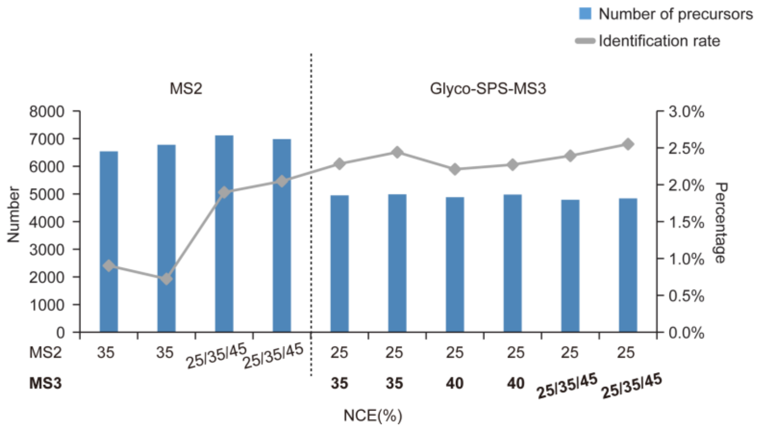

Supplementary figure 9. Number of total triggered precursors and the spectra identification rates using IgM digests in standard MS2 or Glyco-SPS-MS3 methods with different NCEs. Source data are provided as a Source Data file.

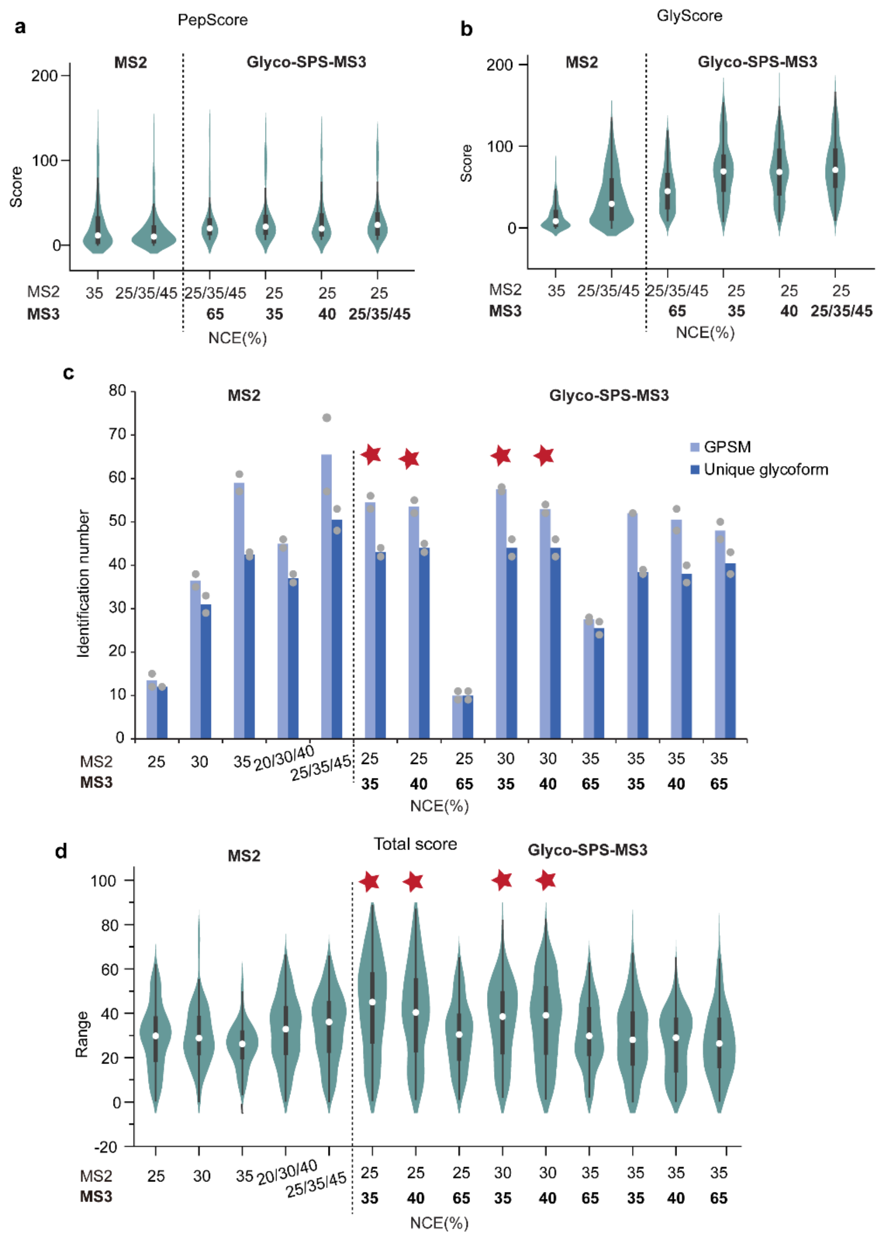

Supplementary figure 10. Optimization of NCE settings for Glyco-SPS-MS3. (a,b) Violin plots showed the peptide score (a) and glycan sore (b) of IgM GPSMs obtained by MS2 or Glyco-SPS-MS3 method with different NCEs in **Fig. 2c,d.** Another preliminary experiment showed the number of average GPSMs and total unique glycoforms (c) and distribution of total scores of GPSMs (d) by MS2 or Glyco-SPS-MS3 with different NCEs from two replicate LC-MS/MS analyses of IgM digests on Lumos. Red stars highlight the methods of choice. Numbers of data points used in panel a and b are (from left to right): 336, 504, 188, 233, 219, and 227. Numbers of data points used in panel d are (from left to right): 148, 146, 150, 149, 162, 137, 132, 130, 144, 151, 138, 146, 155, and 152. Violin plots show the median (white dots), first and third quartiles (box edges) and 1.5 × the interquartile range (whiskers). Source data are provided as a Source Data file.

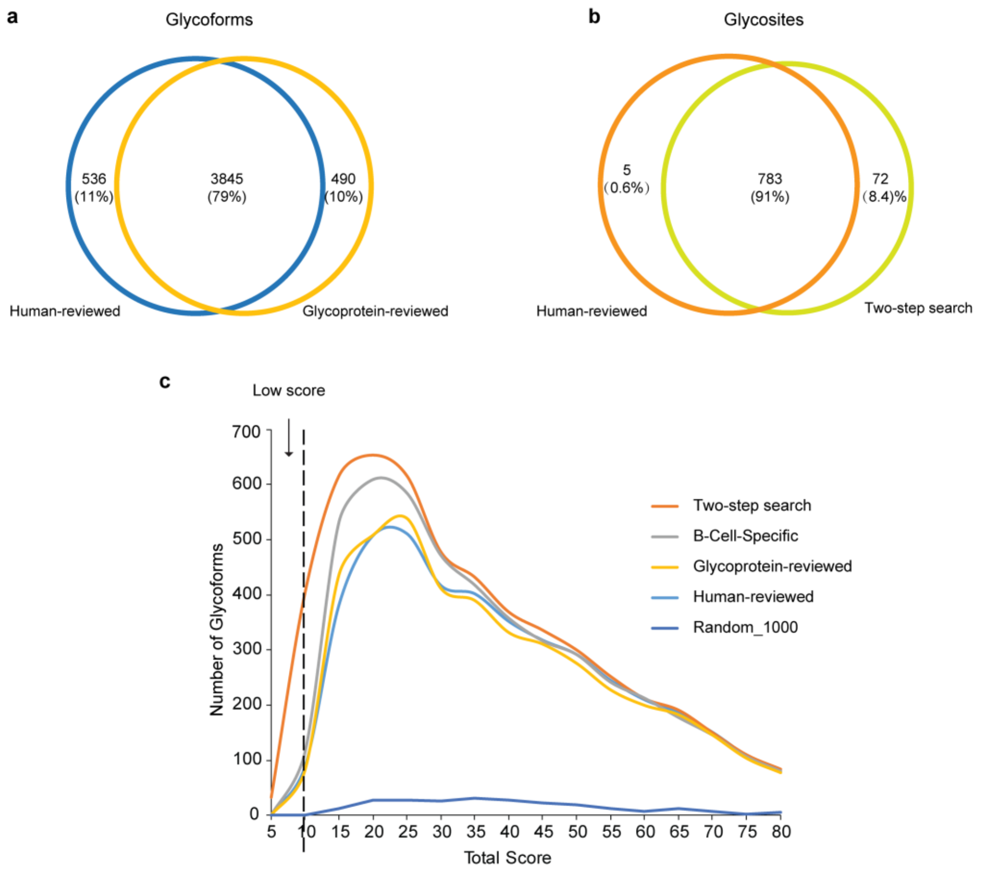

Supplementary figure 11: The effects on the numbers of glycopeptide identification by searching against various refined protein databases. We showed the overlap of identified glycoforms (a) and glycosites (b) resulted from different protein databases in Venn diagrams. (c) Aligned score distributions of the glycoforms identified by using different protein databases. Source data are provided as a Source Data file.

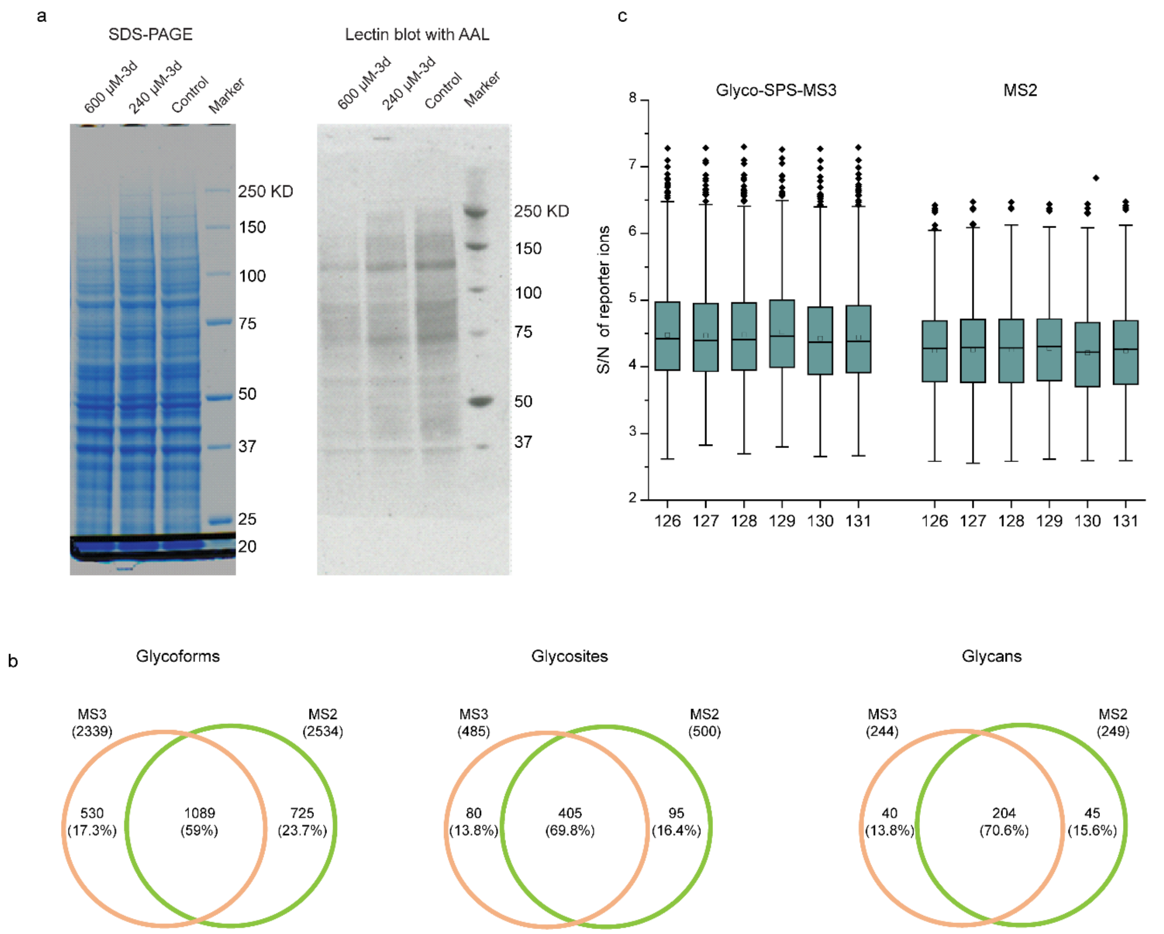

Supplementary figure 12. **Reduced fucosylation in 2FF-treated DG75 cells revealed by lectin blotting and comparison of identifications from DG75 cells using either MS2 or Glyco-SPS-MS3.** (a) DG75 cells were treated with 2FF at the final concentrations of 240 µM or 600 µM for 3 days or mock-treated (control). After lysis, equal protein amounts extracted from different treatments were loaded on two duplicate SDS-PAGEs. One was stained with coomassie brilliant blue (left), and the other one was blotted against AAL lectin (right). No replicate experiments were performed. (b) Venn diagrams showing the overlaps of identified glycoforms (left), glycosites (middle), and glycans (right) using either the MS2 (pink) or the Glyco-SPS-MS3 (green) methods. (c) Box plots showing the reporter ion signal to noise (S/N) detected by either the Glyco-SPS-MS3 (left) or the MS2 (right). Boxplots show the median (centerline), mean (squares), first and third quartiles (box edges) and 1.5 × the interquartile range (whiskers) and outliers. Numbers of data points in panel c are (from left to right): 4409, 4407, 4399, 4406, 4393, 4403, 3444, 3440, 3441, 3442, 3438, and 3434. Source data are provided as a Source Data file.

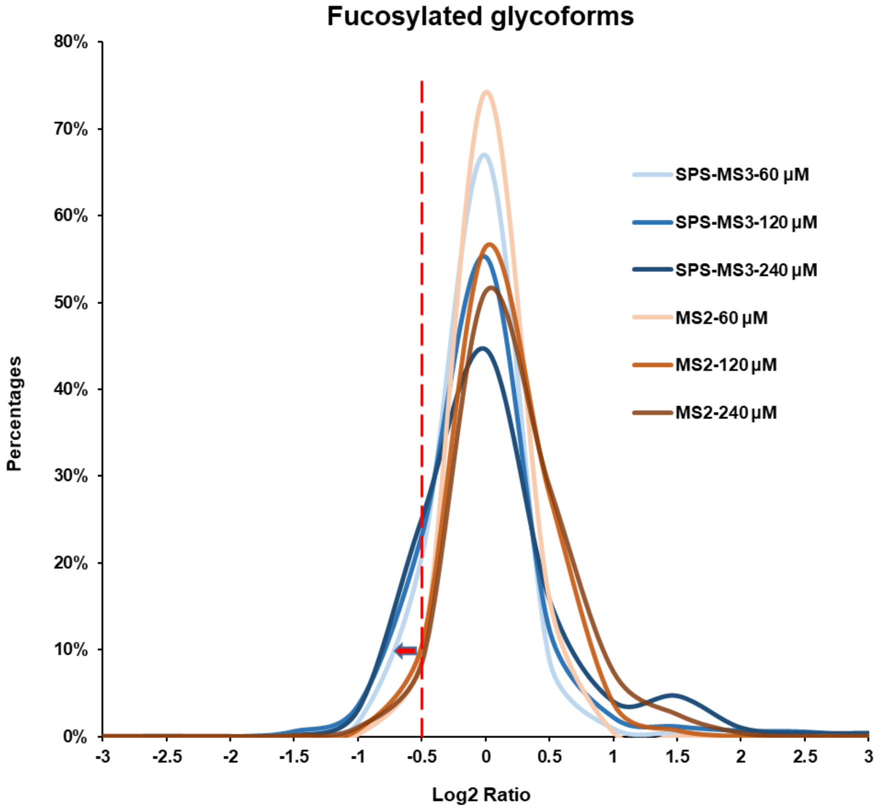

Supplementary figure 13. Glyco-SPS-MS3 determined modestly decreased fucosylation in DG75 cells treated with lower concentrations of 2FF. Ratio distributions of glycoforms quantified via the Glyco-SPS-MS3 and the MS2 method upon treatment with lower 2FF concentrations (60 μM, 120 μM and 240 μM) were aligned. Different 2FF concentrations were color-coded as shown in the figure. Source data are provided as a Source Data file.

Pearson Correlation

60 µM-R1-1	NaN					
60 µM-R1-2	**0.59**	NaN				
60 µM-R1-3	**0.67**	**0.68**	NaN			
60 µM-R2-1	**0.53**	**0.55**	**0.54**	NaN		
60 µM-R2-2	**0.52**	**0.58**	**0.55**	**0.88**	NaN	
60 µM-R2-3	**0.56**	**0.59**	**0.59**	**0.89**	**0.93**	NaN
	60 µM-R1			60 µM-R2		

120 µM-R1-1	NaN					
120 µM-R1-2	**0.84**	NaN				
120 µM-R1-3	**0.83**	**0.85**	NaN			
120 µM-R2-1	**0.40**	**0.43**	**0.42**	NaN		
120 µM-R2-2	**0.33**	**0.37**	**0.38**	**0.89**	NaN	
120 µM-R2-3	**0.41**	**0.43**	**0.42**	**0.90**	**0.89**	NaN
	120 µM-R1			120 µM-R2		

240 µM-R1-1	NaN					
240 µM-R1-2	**0.85**	NaN				
240 µM-R1-3	**0.85**	**0.89**	NaN			
240 µM-R2-1	**0.74**	**0.79**	**0.77**	NaN		
240 µM-R2-2	**0.77**	**0.83**	**0.82**	**0.94**	NaN	
240 µM-R2-3	**0.80**	**0.85**	**0.84**	**0.95**	**0.98**	NaN
	240 µM-R1			240 µM-R2		

480 µM-R1-1	NaN					
480 µM-R1-2	**0.70**	NaN				
480 µM-R1-3	**0.72**	**0.79**	NaN			
480 µM-R2-1	**0.58**	**0.59**	**0.58**	NaN		
480 µM-R2-2	**0.56**	**0.60**	**0.59**	**0.94**	NaN	
480 µM-R2-3	**0.56**	**0.61**	**0.58**	**0.93**	**0.96**	NaN
	480 µM-R1			480 µM-R2		

600 µM-R1-1	NaN					
600 µM-R1-2	**0.72**	NaN				
600 µM-R1-3	**0.74**	**0.82**	NaN			
600 µM-R2-1	**0.65**	**0.71**	**0.68**	NaN		
600 µM-R2-2	**0.63**	**0.72**	**0.70**	**0.95**	NaN	
600 µM-R2-3	**0.66**	**0.72**	**0.70**	**0.96**	**0.98**	NaN
	600 µM-R1			600 µM-R2		

0.5 1

Supplementary figure 14. An overview of Pearson correlations between replicates. Each biological replicate contains technical triplicates. The first biological replicate is marked in blue, and the second in green. Source data are provided as a Source Data file.

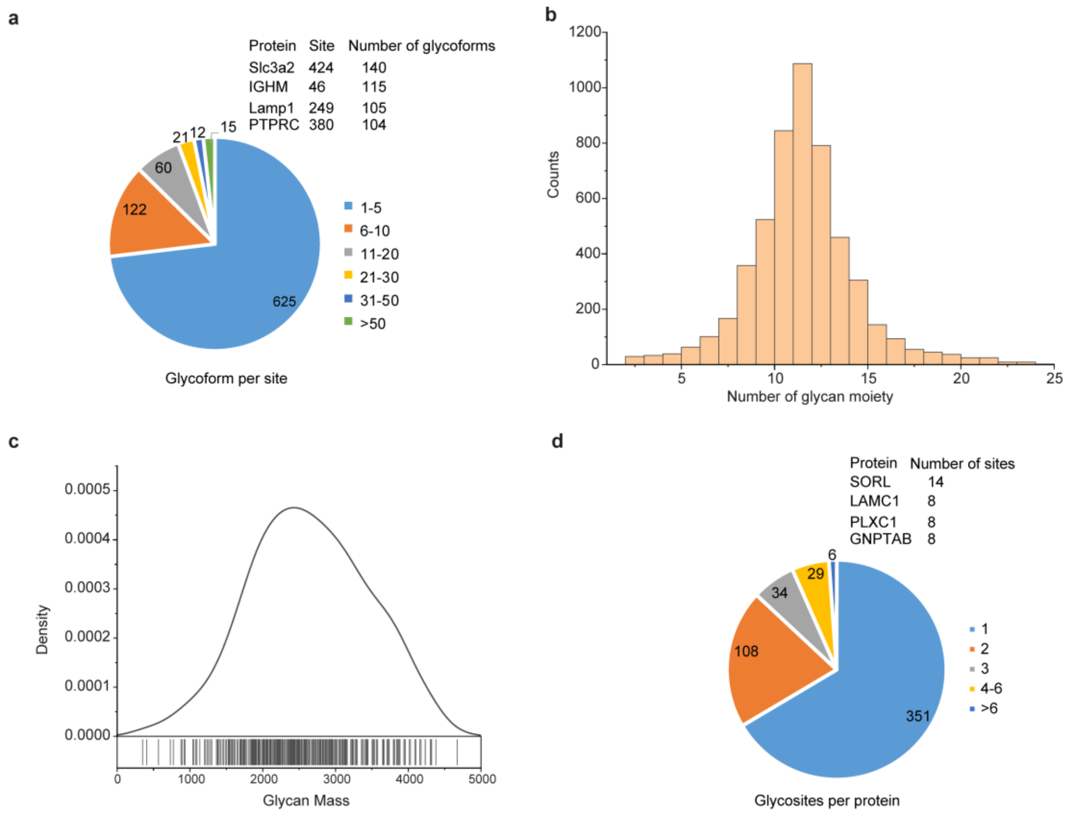

Supplementary figure 15. **Heterogeneity of glycosylation detected in DG75 cells.** (a) Identified glycosites were grouped according to the numbers of observed glycoforms on them, and are shown as a pie chart. Different groups are color-coded as shown in the figure. The glycosites bearing the highest numbers of glycoforms are highlighted. (b) The distribution of the number of monosaccharide moiety per identified glycan. (c) The distribution of glycan masses. (d) Similar to (a) but showing the number of identified glycosites per protein. Glycoproteins with the highest number of identified glycosites are highlighted. Source data are provided as a Source Data file.

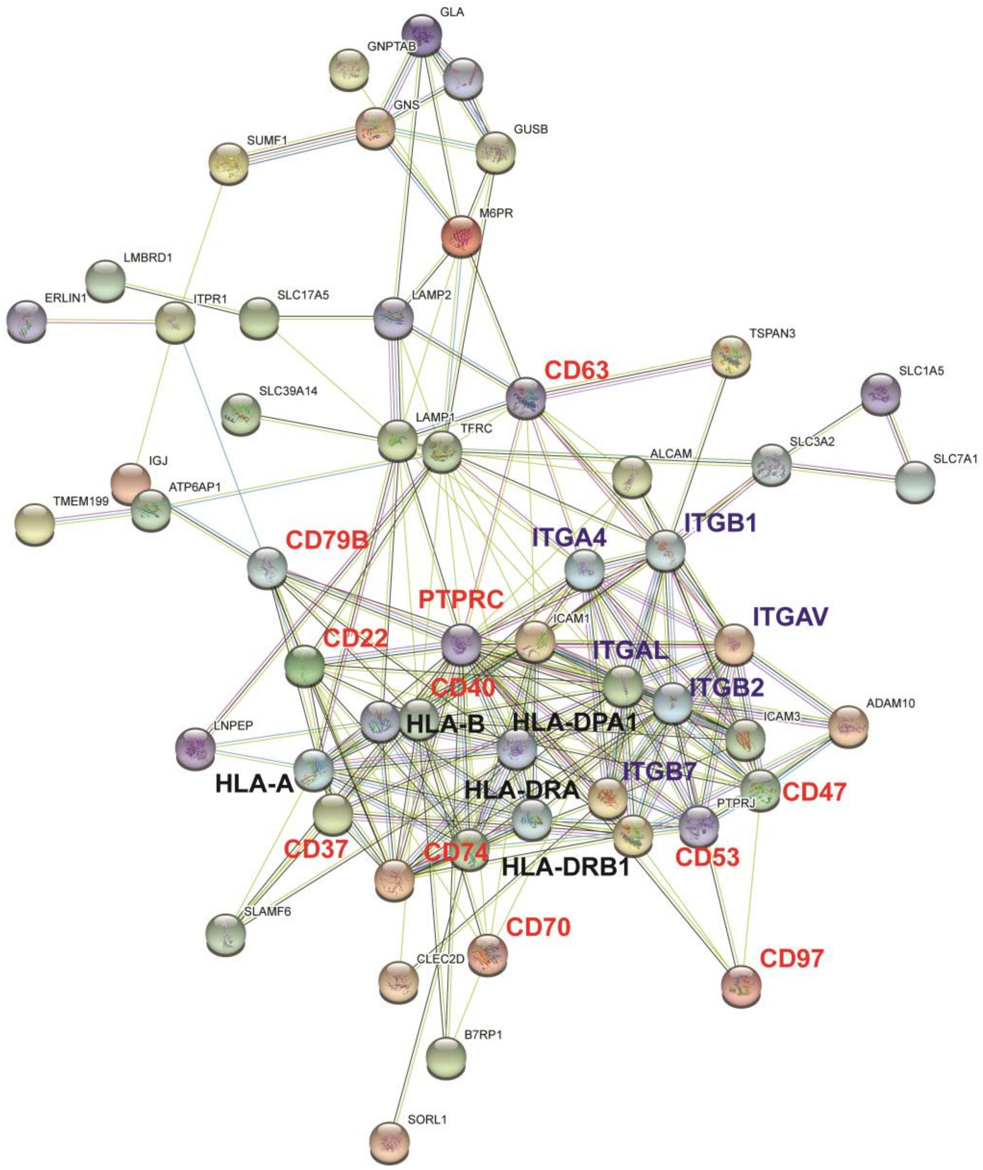

Supplementary figure 16. **STRING interaction network of proteins with differentially regulated glycosylation in 2FF-treated DG75 cells.** CD molecules, integrins and MHC molecules are marked with different colors.

Supplementary Table 1. Optimization of MS parameters in Glyco-SPS-MS3 method.

**Parameters**	**Settings**	**Optimal**	**Figures**
Detector in MS2	Orbitrap, ion trap	Orbitrap	**Supp. Fig. 5**
Fragmentation in MS2	HCD, CID	HCD	**Supp. Fig. 5**
HCD NCE in MS2	Single energy: 25, 30, 35<br>sNCE : 20\30\40, 25\35\45	sNCE-25\35\45	**Fig. 2;**<br>**Supp. Fig. 9**
HCD NCE in MS2 and MS3	1. MS2 (single energy)_MS3 (single energy): 25_35, 25_40, 25_65, 30_35, 30_40, 30_65, 35_35, 35_40, 35_65;<br>2. MS2 (sNCE)_MS3 (single energy): 25\35\45_65;<br>3. MS2 (single energy)_MS3 (sNCE): 25_25\35\45;	25_35; 25_40;	
Injection time in MS2	100 ms; 250 ms; 500 ms	500 ms	**Supp. Fig. 7**
Injection time allocation in MS2 and MS3 (ms)	MS2_MS3: 150_350; 200_300; 250_250; 300_200; 350_150	150_350	
AGC	$5e^4$; e^5; $5e^5$	$5e^5$	
Notches	3; 5; 10	10	**Supp. Fig. 6**

Supplementary Table 2: Suggested MS settings for Glyco-SPS-MS3 for TMT-labelled glycopeptide analysis.

		MS2	**Glyco-SPS-MS3**
MS1	MS instrument	**Orbitrap Fusion or Lumos**	
	Detector Type	**Orbitrap**	
	Orbitrap Resolution	**120 k**	
	Mass Range (m/z)	**350-2000**	
	Maximum injection time (ms)	**50**	
	AGC target	**$5e^5$**	
	RF Lens	**60%**	
	DataType	**Profile**	
	Precursor selection range (m/z)	**700-2000**	
MS2	Isolation mode	Quadrupole	**Quadrupole**
	Isolation window (m/z)	2	**2**
	Scan range mode	Auto normal	**Auto normal**
	First mass (m/z)	120	**132**
	Activation type	HCD	**HCD**
	Normalized collision energy (%)	25/35/45	**25**
	Detector type	Orbitrap	**Orbitrap**
	Orbitrap resolution	15 K	**15 K**
	Maximum injection time (ms)	500	**150**
	AGC target	$5e^5$	**$5e^5$**
	Data type	Profile	**Profile**
	Precursor selection range (m/z)		**700-2000**
MS3	Number of Notches		**10**
	Isolation mode		**Quadrupole**
	Isolation window (m/z)		**2**
	MS2 isolation window (m/z)		**2**
	First mass (m/z)		**120**
	Scan range mode		**Auto normal**
	Activation type		**HCD**
	Collision energy (%)		**35**
	Detector type		**Orbitrap**
	Orbitrap resolution		**15 K**
	Maximum injection time (ms)		**350**
	AGC target		**$5e^5$**

Supplementary References

1. Nikolov, M., Schmidt, C. & Urlaub, H. Quantitative mass spectrometry-based proteomics: an overview. *Methods Mol. Biol.* **893**, 85-100 (2012).
2. Park, G.W. et al. Integrated GlycoProteome Analyzer (I-GPA) for Automated Identification and Quantitation of Site-Specific N-Glycosylation. *Sci. Rep.* **6**, 21175 (2016).
3. Mayampurath, A. et al. Label-free glycopeptide quantification for biomarker discovery in human sera. *J. Proteome Res.* **13**, 4821-4832 (2014).
4. Rebecchi, K.R., Wenke, J.L., Go, E.P. & Desaire, H. Label-free quantitation: a new glycoproteomics approach. *J. Am. Soc. Mass Spectrom.* **20**, 1048-1059 (2009).
5. Pan, K.-T., Chen, C.-C., Urlaub, H. & Khoo, K.-H. Adapting Data-Independent Acquisition for Mass Spectrometry-Based Protein Site-Specific N-Glycosylation Analysis. *Anal. Chem.* **89**, 4532-4539 (2017).
6. Ye, Z., Mao, Y., Clausen, H. & Vakhrushev, S.Y. Glyco-DIA: a method for quantitative O-glycoproteomics with in silico-boosted glycopeptide libraries. *Nat. Methods* (2019).
7. Ong, S.E. et al. Stable isotope labeling by amino acids in cell culture, SILAC, as a simple and accurate approach to expression proteomics. *Mol. Cell. Proteomics* **1**, 376-386 (2002).
8. Rauniyar, N. & Yates, J.R. Isobaric labeling-based relative quantification in shotgun proteomics. *J. Proteome Res.* **13**, 5293-5309 (2014).
9. Budnik, B., Levy, E., Harmange, G. & Slavov, N. SCoPE-MS: mass spectrometry of single mammalian cells quantifies proteome heterogeneity during cell differentiation. *Genome Biol.* **19**, 161 (2018).
10. Robinson, R.C., Poulsen, N.A. & Barile, D. Multiplexed bovine milk oligosaccharide analysis with aminoxy tandem mass tags. *PLoS One* **13**, e0196513 (2018).
11. Sun, S. et al. Comprehensive analysis of protein glycosylation by solid-phase extraction of N-linked glycans and glycosite-containing peptides. *Nat. Biotechnol.* **34**, 84-88 (2016).
12. Stadlmann, J. et al. Comparative glycoproteomics of stem cells identifies new players in ricin toxicity. *Nature* **549**, 538-542 (2017).
13. Lee, H.J. et al. Abundance-ratio-based semiquantitative analysis of site-specific N-linked glycopeptides present in the plasma of hepatocellular carcinoma patients. *J. Proteome Res.* **13**, 2328-2338 (2014).
14. Zhu, H., Qiu, C., Ruth, A.C., Keire, D.A. & Ye, H. A LC-MS All-in-One Workflow for Site-Specific Location, Identification and Quantification of N-/O- Glycosylation in Human Chorionic Gonadotropin Drug Products. *AAPS J* **19**, 846-855 (2017).
15. Savitski, M.M. et al. Measuring and managing ratio compression for accurate iTRAQ/TMT quantification. *J. Proteome Res.* **12**, 3586-3598 (2013).
16. Seddon, A.M., Curnow, P. & Booth, P.J. Membrane proteins, lipids and detergents: not just a soap opera. *Biochim. Biophys. Acta* **1666**, 105-117 (2004).
17. Ilavenil, S. et al. Removal of SDS from biological protein digests for proteomic analysis by mass spectrometry. *Proteome Sci* **14**, 11 (2016).
18. Kachuk, C., Faulkner, M., Liu, F. & Doucette, A.A. Automated SDS Depletion for Mass Spectrometry of Intact Membrane Proteins though Transmembrane Electrophoresis. *J. Proteome Res.* **15**, 2634-2642 (2016).
19. Xia, S. et al. Integrated SDS removal and protein digestion by hollow fiber membrane based device for SDS-assisted proteome analysis. *Talanta* **141**, 235-238 (2015).
20. Yu, Y.Q., Gilar, M. & Gebler, J.C. A complete peptide mapping of membrane proteins: a novel surfactant aiding the enzymatic digestion of bacteriorhodopsin. *Rapid Commun. Mass Spectrom.* **18**, 711-715 (2004).
21. Hughes, C.S. et al. Ultrasensitive proteome analysis using paramagnetic bead technology. *Mol. Syst. Biol.* **10**, 757 (2014).
22. Tanveer S. B. et al. Protein aggregation capture on microparticles enables multipurpose proteomics sample preparation. *Mol. Cell. Proteomics* **18**, 1027–1035 (2019).
23. Wuhrer, M., Catalina, M.I., Deelder, A.M. & Hokke, C.H. Glycoproteomics based on tandem mass spectrometry of glycopeptides. *J. Chromatogr. B* **849**, 115-128 (2007).
24. Liu, M.Q. et al. pGlyco 2.0 enables precision N-glycoproteomics with comprehensive quality control and one-step mass spectrometry for intact glycopeptide identification. *Nat. Commun.* **8**, 438 (2017).
25. Riley, N.M., Hebert, A.S., Westphall, M.S. & Coon, J.J. Capturing site-specific heterogeneity with large-scale N-glycoproteome analysis. *Nat. Commun.* **10**, 1311 (2019).
26. Zeng, W.F. et al. pGlyco: a pipeline for the identification of intact N-glycopeptides by using HCD-and CID-MS/MS and MS3. *Sci. Rep.* **6**, 25102 (2016).
27. Wu, S.W., Pu, T.H., Viner, R. & Khoo, K.H. Novel LC-MS(2) product dependent parallel data acquisition function and data analysis workflow for sequencing and identification of intact glycopeptides. *Anal. Chem.* **86**, 5478-5486 (2014).
28. Hu, H., Khatri, K. & Zaia, J. Algorithms and design strategies towards automated glycoproteomics analysis. *Mass Spectrom. Rev.* **36**, 475-498 (2017).

29. Nilsson, J. et al. Enrichment of glycopeptides for glycan structure and attachment site identification. *Nat. Methods* **6**, 809-811 (2009).
30. Ting, L., Rad, R., Gygi, S.P. & Haas, W. MS3 eliminates ratio distortion in isobaric multiplexed quantitative proteomics. *Nat. Methods* **8**, 937-940 (2011).
31. McAlister, G.C. et al. MultiNotch MS3 enables accurate, sensitive, and multiplexed detection of differential expression across cancer cell line proteomes. *Anal. Chem.* **86**, 7150-7158 (2014).
32. Kalli, A. & Hess, S. Effect of mass spectrometric parameters on peptide and protein identification rates for shotgun proteomic experiments on an LTQ-orbitrap mass analyzer. *Proteomics* **12**, 21-31 (2012).
33. Kovalchik, K.A. et al. RawTools: Rapid and Dynamic Interrogation of Orbitrap Data Files for Mass Spectrometer System Management. *J. Proteome Res.* **18**, 700-708 (2019).
34. Holman, J.D., Tabb, D.L. & Mallick, P. Employing ProteoWizard to Convert Raw Mass Spectrometry Data. *Curr. Protoc. Bioinformatics* **46**, 13 24 11-19 (2014).
35. Yuan, Z.F. et al. pParse: a method for accurate determination of monoisotopic peaks in high-resolution mass spectra. *Proteomics* **12**, 226-235 (2012).
36. Bern, M., Kil, Y.J. & Becker, C. Byonic: advanced peptide and protein identification software. *Curr. Protoc. Bioinformatics* **40**, 13-20 (2012).
