# Supplementary material for: A streamlined pipeline for multiplexed quantitative site-specific N-glycoproteomics
Source: Nat Commun. 2020 Oct 19;11:5268. doi: 10.1038/s41467-020-19052-w (PMC7572468; doi:10.1038/s41467-020-19052-w)
Supplement: Supplementary file 3 — Description of Additional Supplementary Files [file 41467_2020_19052_MOESM3_ESM.docx]

**Description of Additional Supplementary Files**

File Name: Supplementary data 1

Description: Different protein databases compared for the glycopeptide data processing

using GlycoBinder.

File Name: Supplementary data 2

Description: Total quantified glycoforms using MS2 and Glyco-SPS-MS3 method in

Burkitts lymphoma cells treated with 2FF.

File Name: Supplementary data 3

Description: Total quantified glycosites using MS2 and Glyco-SPS-MS3 method in Burkitts

lymphoma cells treated with 2FF.

File Name: Supplementary data 4

Description: Total quantified glycans using MS2 and Glyco-SPS-MS3 method in Burkitts

lymphoma cells treated with 2FF.

File Name: Supplementary data 5

Description: Functional annotation and classification of the 2FF-affected glycoproteins for

Sunburst.

File Name: Supplementary data 6

Description: Quantitative proteome in Burkitts lymphoma cells treated with 2FF.

File Name: Supplementary data 7

Description: Description of all .raw data and associated figures.
