# Supplementary material for: A streamlined pipeline for multiplexed quantitative site-specific N-glycoproteomics
Source: Nat Commun. 2020 Oct 19;11:5268. doi: 10.1038/s41467-020-19052-w (PMC7572468; doi:10.1038/s41467-020-19052-w)
Supplement: Supplementary file 11 — Reporting Summary [file 41467_2020_19052_MOESM11_ESM.pdf]

nature research

Corresponding author(s): Kuan-Ting Pan, Henning Urlaub

Last updated by author(s): Sep 9, 2020

Reporting Summary

Nature Research wishes to improve the reproducibility of the work that we publish. This form provides structure for consistency and transparency in reporting. For further information on Nature Research policies, see Authors & Referees and the Editorial Policy Checklist .

Statistics

For all statistical analyses, confirm that the following items are present in the figure legend, table legend, main text, or Methods section.

n/a	Confirmed	
☐	☒	The exact sample size (*n*) for each experimental group/condition, given as a discrete number and unit of measurement
☐	☒	A statement on whether measurements were taken from distinct samples or whether the same sample was measured repeatedly
☐	☒	The statistical test(s) used AND whether they are one- or two-sided<br>*Only common tests should be described solely by name; describe more complex techniques in the Methods section.*
☒	☐	A description of all covariates tested
☐	☒	A description of any assumptions or corrections, such as tests of normality and adjustment for multiple comparisons
☐	☒	A full description of the statistical parameters including central tendency (e.g. means) or other basic estimates (e.g. regression coefficient) AND variation (e.g. standard deviation) or associated estimates of uncertainty (e.g. confidence intervals)
☐	☒	For null hypothesis testing, the test statistic (e.g. *F*, *t*, *r*) with confidence intervals, effect sizes, degrees of freedom and *P* value noted<br>*Give P values as exact values whenever suitable.*
☒	☐	For Bayesian analysis, information on the choice of priors and Markov chain Monte Carlo settings
☐	☒	For hierarchical and complex designs, identification of the appropriate level for tests and full reporting of outcomes
☒	☐	Estimates of effect sizes (e.g. Cohen's *d*, Pearson's *r*), indicating how they were calculated

Our web collection on statistics for biologists contains articles on many of the points above.

Software and code

Policy information about availability of computer code

Data collection	All data were acquired on Thermo Scientific Orbitrap Fusion or Lumos Tribrid MS.
Data analysis	For intact glycopeptide identification and quantification, .raw files were processed via GlycoBinder.<br>GlycoBinder is written in R (version 3.5.0.) and available on GitHub (https://github.com/IvanSilbern/GlycoBinder). It relies on the usage of external tools.<br>1.RawTools, version 2.0.2 [https://github.com/kevinkovalchik/RawTools]<br>2.msconvert (ProteoWizard), version 3.0.19262 (0a01c36ac) [https://github.com/ProteoWizard/pwiz]<br>3.pParse, version 2.0.8 [http://pfind.net/software/pParse/index.html]<br>4.pGlyco, version 2.2.0 [http://pfind.net/software/pGlyco/index.html]<br>Byonic, version 2.8.2<br>Microsoft excel 2016<br>OriginPro 2020 (9.7.0.185).<br>Thermo Proteome Discoverer, version 2.2<br>STRING (version 11.0)

For manuscripts utilizing custom algorithms or software that are central to the research but not yet described in published literature, software must be made available to editors/reviewers. We strongly encourage code deposition in a community repository (e.g. GitHub). See the Nature Research guidelines for submitting code & software for further information.

Data

Policy information about availability of data

All manuscripts must include a data availability statement. This statement should provide the following information, where applicable:

- Accession codes, unique identifiers, or web links for publicly available datasets
- A list of figures that have associated raw data
- A description of any restrictions on data availability

The mass spectrometry proteomics data have been deposited to the ProteomeXchange Consortium via the PRIDE partner repository with the dataset identifier PXD018349. Source data are provided with this paper. All protein sequence database used in this study, including Human-reviewed, Glycoprotein-reviewed, B-cell-specific, and Random-1000 are listed in Supplementary Data 1

Field-specific reporting

Please select the one below that is the best fit for your research. If you are not sure, read the appropriate sections before making your selection.

[x] Life sciences [ ] Behavioural & social sciences [ ] Ecological, evolutionary & environmental sciences

For a reference copy of the document with all sections, see nature.com/documents/nr-reporting-summary-flat.pdf

Life sciences study design

All studies must disclose on these points even when the disclosure is negative.

Sample size	No statistics method was used to pre-determine sample size. In all experiments, only high confident spectrum-matches with FDR<2% or with stringent Byonic scores were considered. The low technical variability as compared with the large effect size justifies the chosen sample sizes (n >= 3).
Data exclusions	No data are excluded.
Replication	All attempts at replication were successful. For optimization of SugarQuant workflow, two or three technical replicates were performed. For the fucosylation inhibition experiment, two biological replicates were performed. The averaged coefficient of variation (CV) among replicates of each experiment is less than 10%, except the glycopeptide enrichment in figure 1f, where we observe CV above 20%.
Randomization	This study did not involve randomization because only one sample per experiment was used for evaluating and optimizing our workflow. For the experiment of fucosylation inhibition, DG75 cells were separately treated with five doses of 2FF (60 µM, 120 µM, 240 µM, 480 µM, 600µM) or mock-treated (DMSO) for three days. Glycopeptides derived from each sample were labelled with individual TMT6 reagents and mixed before LC-MS/MS analyses.
Blinding	Blinding is not relevant to this study because all samples are known and no human subjective qualitative results were reported.

Reporting for specific materials, systems and methods

We require information from authors about some types of materials, experimental systems and methods used in many studies. Here, indicate whether each material, system or method listed is relevant to your study. If you are not sure if a list item applies to your research, read the appropriate section before selecting a response.

Materials & experimental systems

n/a	Involved in the study	
[x]	[ ]	Antibodies
[ ]	[x]	Eukaryotic cell lines
[x]	[ ]	Palaeontology
[x]	[ ]	Animals and other organisms
[x]	[ ]	Human research participants
[x]	[ ]	Clinical data

Methods

n/a	Involved in the study	
[x]	[ ]	ChIP-seq
[x]	[ ]	Flow cytometry
[x]	[ ]	MRI-based neuroimaging

Eukaryotic cell lines

Policy information about cell lines

Cell line source(s)	DG75 cells (DSMZ no.: ACC 83) were provided by Prof. Dr. Thorsten Zenz at the National Centre for Tumor Diseases (NCT) in Heidelberg, Germany. Daudi cells (DSMZ no.: ACC 78) were purchased from Leibniz Institute DSMZ-German Collection of Microorganisms and Cell Cultures GmbH in Braunschweig, Germany.
Authentication	Both cell lines were authenticated using Multiplex Cell Authentication by Multiplexion (Heidelberg, Germany) as described recently (Castro F, Dirks WG, Fähnrich S, Hotz-Wagenblatt A, Pawlita M, Schmitt M. 2012. High-throughput SNP-based authentication of human cell lines. Int J Cancer. 2012 Jun 15. doi: 10.1002/ijc.27675.). The SNP profiles matched known profiles.
Mycoplasma contamination	Cell lines were regularly tested for mycoplasma according to a PCR protocol published by Uphoff and Drexler (Uphoff CC, Drexler HG. Detection of mycoplasma contaminations. Methods Mol Biol 2005;290:13-23). Only myocoplasma-negative cell lines were used in the study.
Commonly misidentified lines (See ICLAC register)	None of the cell lines used are listed in the ICLAC database.
